# A database of high-resolution MS/MS spectra for lichen metabolites

**DOI:** 10.1038/s41597-019-0305-1

**Published:** 2019-11-28

**Authors:** Damien Olivier-Jimenez, Marylène Chollet-Krugler, David Rondeau, Mehdi A. Beniddir, Solenn Ferron, Thomas Delhaye, Pierre-Marie Allard, Jean-Luc Wolfender, Harrie J. M. Sipman, Robert Lücking, Joël Boustie, Pierre Le Pogam

**Affiliations:** 1 CNRS, ISCR (Institut des Sciences Chimiques de Rennes)-UMR 6226, Univ Rennes, F-35000 Rennes, France; 2 CNRS, IETR (Institut d’Électronique et Télécommunications de Rennes)-UMR 6164, Univ Rennes, F-35000 Rennes, France; 3 Département de Chimie, Université de Bretagne Occidentale, F-29238 Brest, France; 4 CNRS, BioCIS (Biomolécules: Conception Isolement et Synthèse)-UMR 8076, Univ Paris-Sud, Université Paris-Saclay, 5, rue J.-B. Clément, F-92290 Châtenay-Malabry, France; 5 School of Pharmaceutical Sciences, EPGL, University of Geneva, University of Lausanne, CMU, 1 Rue Michel Servet, 1211 Geneva 4, Switzerland; 6 Botanischer Garten und Botanisches Museum, Freie Universität Berlin, Königin-Luise-Strasse 6–8, D-14195 Berlin, Germany

**Keywords:** Metabolomics, Natural products, Mass spectrometry, Secondary metabolism, Small molecules

## Abstract

While analytical techniques in natural products research massively shifted to liquid chromatography-mass spectrometry, lichen chemistry remains reliant on limited analytical methods, Thin Layer Chromatography being the gold standard. To meet the modern standards of metabolomics within lichenochemistry, we announce the publication of an open access MS/MS library with 250 metabolites, coined LDB for Lichen DataBase, providing a comprehensive coverage of lichen chemodiversity. These were donated by the Berlin Garden and Botanical Museum from the collection of Siegfried Huneck to be analyzed by LC-MS/MS. Spectra at individual collision energies were submitted to MetaboLights (https://www.ebi.ac.uk/metabolights/MTBLS999) while merged spectra were uploaded to the GNPS platform (CCMSLIB00004751209 to CCMSLIB00004751517). Technical validation was achieved by dereplicating three lichen extracts using a Molecular Networking approach, revealing the detection of eleven unique molecules that would have been missed without LDB implementation to the GNPS. From a chemist’s viewpoint, this database should help streamlining the isolation of formerly unreported metabolites. From a taxonomist perspective, the LDB offers a versatile tool for the chemical profiling of newly reported species.

## Abstract (summary)

**Table:** 

Measurement(s)	lichen metabolite
Technology Type(s)	liquid chromatography-tandem mass spectrometry
Factor Type(s)	organism
Sample Characteristic - Organism	lichen

Machine-accessible metadata file describing the reported data: 10.6084/m9.figshare.10265234

## Background & Summary

**Fig. 1:**
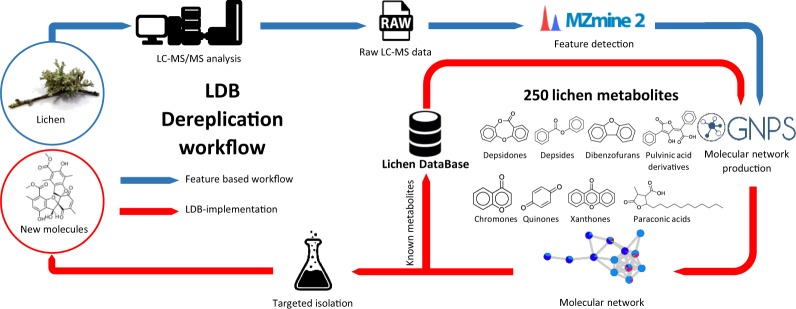
LDB workflow sustained by 250 lichen metabolites as of 07/2019.

In the 19^th^ century, lichens were still considered to be lower plants but around the time the general concept of symbiosis was being developed, they were found to be associations between a fungus and an alga^1,2^. It was soon unveiled that this original lifestyle was accompanied by a specific chemodiversity. As knowledge regarding lichen chemistry progressed, tailored analytical approaches to harness this specific chemistry were developed. Accordingly, during the middle of the twentieth century Asahina and Shibata performed microcrystallizations to visually identify lichens metabolites, and started elucidating their structures^3–5^. TLC was later introduced in an attempt to dereplicate more precisely lichen metabolites and standardized migration solvent mixtures were designed in 1970 by Culberson & Kristinsson^6^, facilitating chemical profiling and identification of species by comparison to co-eluted standards. Although lichen chemicals were historically used as taxonomic markers, the delimitation of species based solely on chemistry was treated with caution. Major compounds and their biosynthetically related minor satellite compounds were however investigated as a means to classify lichens in chemosydromes to better understand the evolution of closely related taxa through their chemistry, although biomolecular data is now more commonplace^7–23^. Data accumulated during the 20^th^ century was summarized in 1996 by Huneck and Yoshimura in their compendium “Identification of Lichen Substances”, comprising spectroscopic data for over 850 lichen chemicals, including TLC retardation factors, infra-red data, electron impact mass spectrometry signals, UV/visible spectra and NMR landmarks^24^. Even though a wide range of analytical techniques have been used to study lichen metabolites^25–29^, the favoured approach among lichenologists remains TLC analysis^30–34^. Because of its accessibility, it is still widely used today along with spot test reactions when describing new lichen species to report the main identified metabolites^35–38^. However, it lacks sensitivity, relies on co-elution with standards, and metabolite identification can be challenging as such approaches are based on comparative identification and not on the generation of proper spectroscopic data. Efforts have been made to update Huneck’s and Yoshimura’s compendium^39–42^ and databases geared towards chemotaxonomy of lichens have arisen, but these are based on a limited number of metabolites, mainly major compounds accessible by TLC detection^43–45^. Aside from their traditional use in chemotaxonomy, lichen metabolites, most notably polyketides, have been known for long to be specifically produced by these organisms and to have a variety of bioactive properties^30,46–52^. Although the last count of lichen metabolites is of about 1050^53^, assessing the chemical diversity sheltered by this privileged biota is rendered especially challenging by the tendency of lichens to accumulate extremely elevated yields of a few major compounds. Moreover, a widely accepted idea is that these symbiotic systems are associated with a rather scarce chemical diversity sustained by a limited number of polyketide scaffolds. Yet, this may be related to a superficial knowledge of the specialized metabolome associated with these fascinating lifeforms. Studying lichens with modern analytical tools shall shed new light on lichen chemistry. Some recent reports demonstrated that some atypical structures are still to be described from lichen sources, as could be seen through the unprecedented skeletons of tsavoenones and sanctis, both obtained last year from Vietnamese *Parmotrema* species^54,55^. To this effect, a molecular networking dereplication pipeline was developed based on the implementation of an in-house MS/MS database (Fig. 1), inspired by the MIADB project spearheaded by Beniddir and co-workers^56^. Pure lichen metabolites from Siegfried Huneck’s chemical library maintained in Berlin, in addition to an in-house chemical library from the laboratory in Rennes, were used to constitute the MS/MS database labelled LDB: Lichen DataBase. It includes MS/MS spectra for 250 small molecules ionized in ESI^−^, ESI^+^ and APCI, representing 23% of the alleged 1050 known lichen metabolites. The most common appendages in lichen metabolites are represented, *i.e*. depsides, depsidones, dibenzofurans, diphenylethers, pulvinic acid derivatives, quinones, xanthones and terpenes (Fig. 1). This database sets a new standard of dereplication in lichen chemistry, regarding both detection sensitivity and structure identification reliability. This new approach also bypasses the mandatory need for holding libraries of standard references since dereplication is based on the online webserver hosted by the GNPS. This data descriptor announces the deposition in public repositories of the LDB on the GNPS^57^ and Metabolights^58,59^ servers.

## Methods

### Sample sources

Samples were prepared from two sources: the Huneck chemical library at the Botanical Garden and Botanical Museum in Berlin (B), and the chemical library from the laboratory in Rennes. The Huneck library contains 1520 numbered and catalogized substances assembled by Siegfried Huneck and collaborators (including Benno Feige), together with additional extracts from his research. A complete list is available from B, compiled by Heidi Kümmerling, Stefanie Schöne, and Harrie Sipman. The chemical library from Rennes contains as of today 144 lichen substances catalogized by the staff of the laboratory.

### Sample preparation

Each of the 250 compounds was solubilized in HPLC-grade methanol at 1 mg/mL and placed into 1.5 mL HPLC vials prior to analysis. Solvents were purchased from Sigma-Aldrich.

### Data acquisition

Samples were analysed using an Agilent 6530 Accurate-Mass Q-TOF hyphenated with a 1260 Agilent Infinity LC system. The column used was a Waters SunFire C_18_ (50 × 4.6 mm, i.d. 3.5 µm) with a flow rate of 0.5 mL/min. Elution solvents used were Milli-Q water +0.1% FA (A) and HPLC-grade acetonitrile +0.1% FA (B) and elution gradient was the following: 0 min at 5% B, 7 min at 100% B, 8 min at 100% B, 9 min at 5% B. Most analytes were ionized by electrospray in negative polarity, xanthones and quinones were additionally ionized in positive mode, and terpenes were ionized exclusively with an APCI source. ESI conditions were set with the capillary temperature at 320 °C, source voltage at 3.5 kV, and a sheath gas flow rate of 10 L/min. Regarding APCI analyses, the corona current was set to 4 *µ*A, the nebulizer pressure was 35 psig and 10 L/min nitrogen flow heated at 350 °C was used for desolvation. Capillary, fragmentor and skimmer voltages were set to 3500 V, 175 V and 65 V respectively. There were four scan events: negative or positive MS, window from *m/z* 100–1200, then three data-dependent MS/MS scans of the first, second, and third most intense ions from the first scan event. MS/MS settings were the following: three collision energies for the negative mode (10, 25, 40 eV, and additionally 2.5, 5, and 7.5 eV for depsides), three for the ESI+ and APCI modes (5, 20 and 35 eV), default charge of 1, isolation width of *m/z* 2. Purine (C_5_H_4_N_4_, *m/z* 121.050873 (positive)), trifluoroacetic acid (CF_3_CO_2_H, *m/z* 112.98559, negative) and HP-0921 (hexakis(*1H*, *1H*, *3H*-tetrafluoropropoxy)-phosphazene C_18_H_18_F_24_N_3_O_6_P_3_, *m/z* 922.009798 (positive), 1033.988109 (negative, trifluoroacetate adduct) were used as internal lock masses. Full scans were acquired at a resolution of 10 000 (*m/z* 922) and 4000 (*m/z* 121) (positive polarity) and 10 000 (*m/z* 1033) and 4 800 (*m/z* 112).

### Database constitution

309 files in the d agilent format were thus generated and converted in an mzXML format using the MSConvert^60^ module from Proteowizard^61,62^. Raw converted data were then treated using a custom script in the R 3.6.0 language^63^ with the MSnBase package^64^ to isolate MS/MS spectra at each collision energy for the given metabolite and save them as a one scan mzXML file. Additionally, a merged MS/MS spectrum was generated as a one scan mzXML file by averaging previously generated spectra for a given compound. Merged mzXML files were assembled in a single mgf file using a private online workflow provided by the GNPS platform and uploaded with a metadata file as an open access MS/MS database (available at https://gnps.ucsd.edu/ProteoSAFe/gnpslibrary.jsp?library=LDB_POSITIVE for the positive mode spectra, and https://gnps.ucsd.edu/ProteoSAFe/gnpslibrary.jsp?library=LDB_NEGATIVE for the negative spectra). Each spectrum was manually curated to assess the quality of the fragmentation and the identity of fragmented metabolites.

### Database description and use in molecular network-based dereplication

**Fig. 2:**
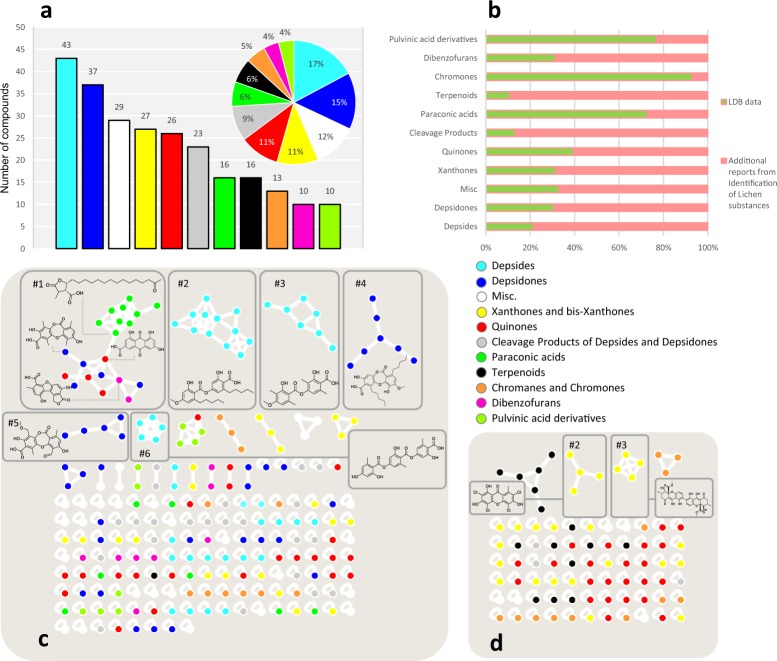
LDB metrics and characteristics. Metabolites are classified according to Huneck and Yoshimura^24^ and the Misc. class represents acids, aliphatic and cycloaliphatic compounds, diphenylethers, mycosporines, naphthopyranes, *N*-containing compounds, polyols, monosaccharides and carbohydrates. Panel a: Number of representatives for each metabolite class and their relative proportions. Panel b: Proportion of metabolites covered for each class. Panels c and d: molecular networks using respectively the negative and positive mode spectra from the lichen metabolites of the LDB as input with a cosine similarity score cut-off of 0.6. Main clusters are numbered and a representative structure of the cluster is displayed.

The LDB contains 309 merged MS/MS spectra from 250 lichen metabolites ionized in Negative Ion mode ESI-MS (NI-ESI) (226 spectra), Positive Ion mode ESI-MS (PI-ESI) (68 spectra) and APCI (15 spectra). Additionally, the 1011 MS/MS spectra at individual collision energies are available on MetaboLights. The available standards cover a huge majority of the scaffolds reported in the 1996 Huneck and Yoshimura compendium (Fig. 2a,b) and can therefore be expected to provide a valuable information for lichen chemical profiling given the high degree of structural recurrence within these organisms. A further criterion for database convenience assessment deals with the ability to provide general data acquisition that encompass as wide as possible a diversity of the metabolites it comprises. The occurrence of acidic functions within a vast majority of lichen metabolites was incentive for their analyses in negative-ion mode, consistently with former reports^25^. While the main analysis parameters (*viz*. 10/25/40 eV collisions energies) afforded convenient tandem mass spectra for most structures investigated herein, two specific cases required further acquisition settings to be applied. At first, depsides underwent an extensive fragmentation under the aforementioned conditions. To remedy, further analyses using lower collision energies were carried out (*i.e*. 2.5/5/7.5 eV) on this specific structural class, which afforded overall better results than the former parameters. All six spectra related to depsides were implemented into the LDB. Besides, one should keep in mind that electrospray ionization mainly facilitates the formation of deprotonated molecules but poorly leads to radical ions^65^. Therefore, in specific molecular environments where phenolic groups can instigate intramolecular hydrogen bonds, their deprotonation is rendered impossible and the molecule is not amenable to NI-ESI-MS detection^66^ without dedicated analytical procedures. As to lichen diversity, this configuration is mostly encountered in γ-pyrone-containing metabolites, *i.e*. quinones, xanthones and chromones, some of which could not be detected upon NI-ESI-MS analyses. However, irrespective of their detected/not-detected status in negative polarities, all of them provided satisfactory MS² spectra in PI-ESI-MS, which should be favoured when analyzing lichen species producing these structural series. At last, terpenes and steroids were analyzed using an APCI source which provides better results than ESI for low to medium-polarity compounds^67^, although Atmospheric Pressure Photo Ionization would probably shift more again towards non-polar compounds. The database is available on GNPS for molecular networking applications. Fig. 2c,d show two molecular networks generated with the spectra from the LDB as input and each node (or molecule) was colored according to the chemical scaffolds they represent, as described by Huneck and Yoshimura^24^ (parameters in Table S1). Generally speaking, the negative-ion mode generated molecular network is bigger due to the much higher number of compounds from the LDB analyzed in this mode, which should prevail in lichenochemistry. The molecular network obtained from the positive-ion mode revealed a more limited clustering, which may be related to the subset of compounds analyzed in this ionization mode also being among the most scattered in negative polarity analyses. Overall, the obtained molecular networks tended to cluster according to compounds’ structural classes. However, each scaffold did not produce a unique and homogenous cluster in the retained settings and, conversely, seemingly-unrelated metabolites sometimes came to cluster together as a consequence of their similar functional groups, thereby being prone to undergoing similar neutral losses. Structural features accounting for the topology of molecular networks are not usually worth being too thoroughly investigated since it exclusively depends on cosine threshold definition, the stringency of which considerably affects the obtained outcome. Nevertheless, some clustering behaviours could be rationalized in a straightforward manner. Illustrative chemical representatives of the discussed clusters are displayed in Fig. 2. As such, an interesting example is that of depsides (Fig. 2c, in cyan) which were split into three major clusters. The cluster #2 contained depsides with three to seven-membered alkyl side-chains, whereas cluster #3 only gathered depsides lacking such lengthy side-chains. At last, cluster #6 comprised tridepsides and a single didepside - lecanoric acid – which can be readily explained by its diaromatic core being encountered in all tridepsides implemented in our LDB collection. A similar side chain-length discrimination within apart clusters could be noted for depsidones (respectively clusters #4 and #5). A last example of rational structure-dependent clustering is that of xanthones that are being clustered differentially depending on their monomeric or dimeric status (Fig. 2d). The southern part of cluster #1 is an example of structurally inhomogeneous-clustering compounds (Fig. 2c), that somehow came to exhibit close-enough fragmentation processes to belong to a same cluster. Such “erratic” structures are represented by quinones (Fig. 2c,d, in red) which produce few fragments in MS/MS and are found mostly as self-looped nodes or related to compounds having similar appendages. Each molecular network presented in this article is available on the GNPS platform: https://gnps.ucsd.edu/ProteoSAFe/status.jsp?task=cc4925fa2ccd43b790b708576f47e7b5 for the negative mode (Fig. 2c) and https://gnps.ucsd.edu/ProteoSAFe/status.jsp?task=c79d748b515b4357a515bcec1435e6a1 for the positive mode (Fig. 2d). Each cluster can be found with the same numbering as in the figure, as well as each node along with their spectra and identifications. To the best of our knowledge, this is the first sizeable MS/MS database of lichen compounds.

## Data Records

MS/MS data of the LDB can be found on the GNPS (merged spectra) and on Metabolights (merged and individual collision energy spectra). It can be accessed through GNPS in the library webpage (https://gnps.ucsd.edu/ProteoSAFe/libraries.jsp), each spectrum having its own accession number, CCMSLIB00004751209 to CCMSLIB00004751434 for negative spectra, CCMSLIB00004751435 to CCMSLIB00004751517 for positive spectra. The spectral collection is also available for download from MetaboLights under the identifier: MTBLS999^59^.

### Metadata

Retention times (RTs) and structures for each metabolite are reported in the Supporting Information. Additional data including, MS acquisition parameters, instrument details, organism, organism part, SMILES and InChI codes, CAS numbers, CHEBI IDs, and chemical formula are available on MetaboLights.

## Technical Validation

### Spectroscopic validation of LDB compounds

Structures of these metabolites were retrieved from the literature and their identity was confirmed by manual curation of the MS/MS spectra by inspecting the parent mass and fragment ions.

### Molecular network-based dereplication of selected lichen acetone extracts

**Fig. 3:**
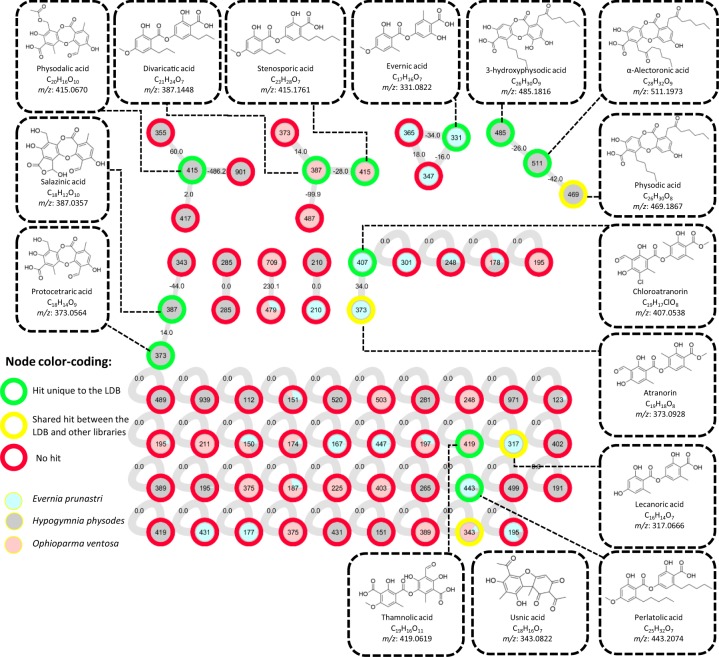
Molecular network generated from the acetone extracts of *Ophioparma ventosa*, *Evernia prunastri* and *Hypogymnia physodes* following the feature-based molecular networking workflow. Nodes with green outer circles represent ions dereplicated solely with the LDB, yellow outer-circles ions are shared by the LDB with other GNPS libraries. Red outer-circles represent nodes that could not be dereplicated automatically. Pie charts inside the nodes represent the proportion with which the ions were observed in each of the three lichens. Identified ions are represented with their structure, name, chemical formulas and theoretical *m/z* values. Edges between nodes are labelled with their difference in mass.

**Fig. 4:**
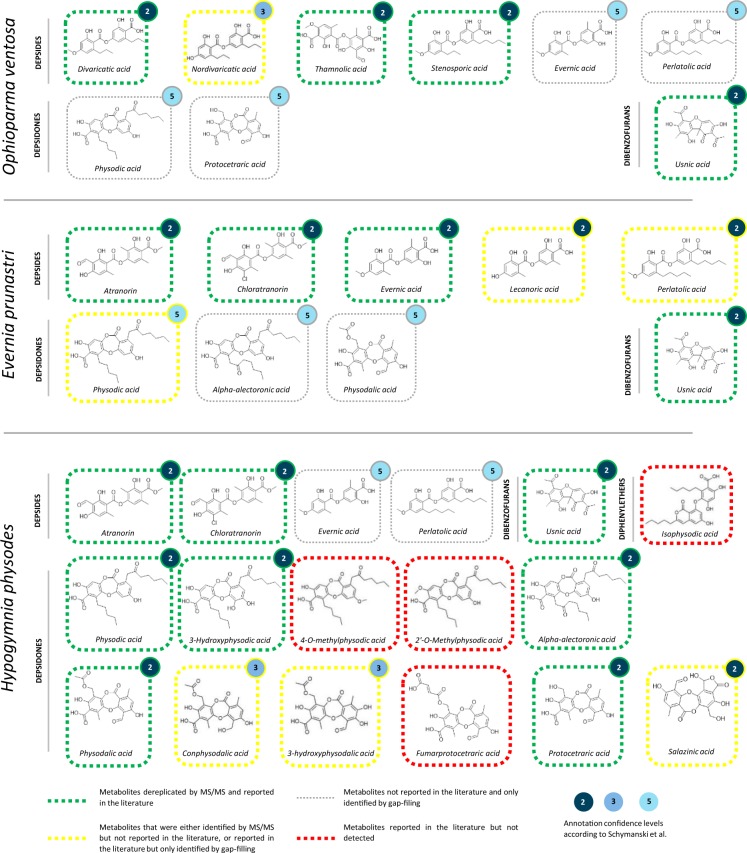
Summary of the molecular networking-based dereplication process of the extracts of *Ophioparma ventosa*, *Evernia prunastri* and *Hypogymnia physodes*. Metabolites dereplicated by MS/MS and reported in the literature for the studied lichen are marked by a green rectangle. Metabolites that were either identified by MS/MS but not reported in the literature, or reported in the literature but only identified by gap-filling are marked by yellow rectangles. Metabolites not reported in the literature and only identified by gap-filling are identified as grey rectangles. Metabolites reported in the literature but not identified in the analysis are shown as red rectangles. All hits are accompanied by a confidence score according to Schymanski *et al*.^69^, from 5 (same exact mass) to 1 (same MS/MS spectrum and RT as the reference standard).

Technical validation was achieved by using the LDB to dereplicate extracts of well documented lichens sampled from the herbarium of Rennes: *Ophioparma ventosa* (JB/14/211), *Evernia prunastri* (JB/13/156) and *Hypogymnia physodes* (JB18/234) (Table S2). Acetone extracts of the lichens were analysed in negative polarity LC-MS and produced files were subjected to an MZmine-GNPS workflow (parameters in Tables S3 and S4). A molecular network was thus produced and dereplication was carried out by three different methods: (i) using only the LDB, (ii) using all GNPS spectral libraries including the LDB, (iii) using all GNPS spectral libraries excluding the LDB. The extent of advantages offered by the LDB was assessed by comparing the hits between the three dereplication methods. The molecular network generated is presented in Fig. 3 with colour-coded nodes depending on the aforementioned dereplication method: (i) green nodes were found exclusively in the LDB, (ii) yellow nodes were identified using all GNPS libraries including the LDB, (iii) red nodes could not be dereplicated. A total of 15 unique molecules were thus dereplicated, with 11 of them being exclusively identified with the LDB, and four being shared with other GNPS libraries. None of the hits were exclusive to other spectral libraries as all were already present in the LDB (Table 1). As annotations generated by the gap-filling function of MZmine^68^ (same *m/z* and RT) are associated with a lesser degree of confidence, the corresponding tags are ranked as 5 according to the widely accepted metabolomics confidence levels defined by Schymanski *et al*.^69^ (Fig. 4). A tentative assignment of some unannotated nodes was performed from the molecular network with the support of the SIRIUS software^70^. In the context of lichenology, molecular networks are particularly useful to emphasize characteristic sets of products, also designated as chemosyndromes. These metabolites are structurally similar because of their biosynthetical interconnections and can therefore be expected to clusterize will form clusters in a molecular network. Such chemosyndromes are often sustained by a major metabolite accompanied by several minor satellite compounds^7^.

**Table 1:** Metabolites annotated by the GNPS libraries.

Source	*m/z*	RT (min)	MS/MS identification	CScore	*m/z* error (ppm)	Shared peaks	Literature
*Hypogymnia physodes*	485.1810	5.95	3-Hydroxyphysodic acid	0.65	10	13	Yes^79,80^
	511.1925	7.33	Alpha-alectoronic acid	0.82	4	19	Yes^79^
	373.0924	8.8	Atranorin	0.85	4	7	Yes^79–82^
	407.0558	8.89	Chloroatranorin	0.87	1	7	Yes^79,81,82^
	331.0828	8.71	Evernic acid	0.56	2	5	No
	443.2071	8.46	Perlatolic acid	0.62	17	5	No
	415.0709	6.59	Physodalic acid	0.93	34	13	Yes^79–82^
	469.1878	8.16	Physodic acid	0.73	15	8	Yes^79–81^
	373.0551	4.93	Protocetraric acid	0.85	18	10	Yes^79,81^
	387.0366	4.29	Salazinic acid	0.88	22	12	No
	343.0823	8.71	Usnic acid	0.80	18	4	Yes^82,83^
*Evernia prunastri*	511.1925	7.33	Alpha-alectoronic acid	0.82	4	19	No
	373.0924	8.8	Atranorin	0.85	4	7	Yes^76,77,84–87^
	407.0558	8.89	Chloroatranorin	0.87	1	7	Yes^76,77,84–88^
	331.0828	8.71	Evernic acid	0.56	2	5	Yes^76,77,84–87^
	317.0660	7.42	Lecanoric Acid	0.75	14	4	No
	443.2071	8.46	Perlatolic acid	0.62	17	5	No
	415.0709	6.59	Physodalic acid	0.93	34	13	No
	469.1878	8.16	Physodic acid	0.73	15	8	Yes^77^
	343.0823	8.71	Usnic acid	0.80	18	4	Yes^76,77,84–87^
*Ophioparma ventosa*	387.1474	9.07	Divaricatic acid	0.84	18	12	Yes^28,73,89^
	331.0828	8.71	Evernic acid	0.56	2	5	No
	443.2071	8.46	Perlatolic acid	0.62	17	5	No
	469.1878	8.16	Physodic acid	0.73	15	8	No
	373.0551	4.93	Protocetraric acid	0.85	18	10	No
	415.1786	9.63	Stenosporic acid	0.87	19	7	Yes^73^
	419.0460	10.4	Thamnolic acid	0.86	42	8	Yes^28,73,89^
	343.0823	8.71	Usnic acid	0.80	18	4	Yes^28,73,89^

### Dereplication of the *Ophioparma ventosa* extract

The chemistry of the crustose lichen *Ophioparma ventosa* was investigated by several research groups over the last decades and its specialized metabolome can be regarded as rather complex. This is related to the presence of some constant metabolites (thamnolic acid, decarboxythamnolic acid, usnic acid, divaricatic acid, haemoventosin and a wealth of further pyranonaphthoquinone pigments)^71,72^ along with some additional compounds, like stenosporic acid, that may occur or not and which are – at least partly – inferred to originate from overgrown lichen species^72–74^. As the *Ophioparma* sample used was rather poor in apothecia, haemoventosin and their related pyronaphthoquinones were not detected. Some recent chemical reports dealing with this lichen species, including thorough phytochemical investigations by our group^28,72^, hinted that the chemistry of this species is quite sharply defined.

Automatic dereplication using the LDB allowed the annotation of four out of the five metabolites expected in negative mode: thamnolic, usnic, stenosporic and divaricatic acids (Table 1). Four other compounds were detected throughout gap-filling strategy (Fig. 4A). None of the latter were previously reported in the literature for *Ophioparma spp*.

These identified metabolites are clustered with some unidentified ones. Divaricatic and stenosporic acids are clustered with two nodes at *m/z* 373.1315 and 487.0743. While the latter can only be assumed to stand for an unknown depside, likely candidates to account for the former could be nordivaricatic acid (4-*O*-demethyldivaricatic acid) previously reported to occur in the frame of both divaricatic and stenosporic acids in several lichens, including *O. ventosa* (Fig. S2)^73^. Another noteworthy cluster for this lichen contains the ion at *m/z* 479.0596, seemingly an usnic acid dibenzofuran-related metabolite (Fig. S3). The joint occurrence of divaricatic acid and stenosporic acid is an illustration of a chemosyndrome, both depsides bearing side chains of moderate length, the latter being a minor satellite compound of the former^73^.

### Dereplication of the *Evernia prunastri* extract

The fruticose lichen *Evernia prunastri* has been thoroughly investigated for its chemical content as it is widely used in fragrance industry. The typical odour of oak moss is related to the hydrolysis of odourless depsides to yield a suite of scentful monoaromatic compounds^75^. Expected compounds as historically described by Culberson^76^ are atranorin, chloratranorin, evernic and usnic acid. Additionally, Joulain and Tabacchi published a critical review of the metabolites reported for *Evernia prunastri* which reached more than 170 structures^75^. As mentioned by the authors, some compounds have to be considered with utmost caution as the sources are sometimes a mixture of plant and lichen material in addition to environmental pollutants resulting in the detection of several petroleum products by GC.

Automatic dereplication allowed the straightforward identification of all four classically described compounds (atranorin, chloratranorin, evernic and usnic acids) in addition to lecanoric acid and perlatolic acid, these two latter metabolites being newly reported in this deeply dug lichen model (Table 1). Physodic acid, previously reported in *Evernia prunastri* was detected by gap-filling^77^. Additional gap-filling-generated metabolites were alpha-alectoronic acid and physodalic acid (Fig. 4B).

Unlabeled nodes clustered with evernic acid include two ions at *m/z* 365.0443 and 347.0764, seemingly sharing a depside scaffold (Figs. S4 and S5). *Evernia prunastri* seems to contain the same usnic acid derivative as *Ophioparma ventosa* at *m/z* 479.0596.

### Dereplication of the *Hypogymnia physodes* extract

This foliose species is known for producing an array of structurally diverse lichen metabolites comprising various orcinol depsides and depsidones differing in the length and hydroxylation status of their side chains^78^. Noteworthy, recent UHPLC-MS² based phytochemical investigations were performed on *H. physodes*^79^, so it can be inferred that a broad coverage of this lichen chemistry is available to evaluate the degree of information conveyed by our current dereplication pipeline. Expected metabolites are usnic acid, atranorin and chloroatranorin, along with a vast range of orcinol depsidones and their derivatives (*viz*. physodic, 3-hydroxyphysodic, 4-*O*-methylphysodic acid, 2′-*O*-methylphysodic acid, isophysodic acid, physodalic, 3-hydroxyphysodalic acid, conphysodalic acid, protocetraric acid and fumarprotocetraric acid), including α-alectoronic acid, a minor metabolite recently revealed to occur within this lichen^79^.

All these metabolites were dereplicated except fumarprotocetraric acid and metabolites absent from the LDB, *i.e*., conphysodalic acid, 4-*O*-methylphysodic acid, 2′-*O*-methylphysodic acid, 3-hydroxyphysodalic acid and isophysodic acid (Table 1). Additionally, salazinic acid, previously unreported in *H. physodes,* was dereplicated. Conphysodalic acid can be linked to a node at *m/z* 417.0833 in the physodalic acid cluster (Figs. 3, S6), as well as 3-hydroxyphysodalic acid as a self-looped node at *m/z* 431.0617. Other level 5 annotations include evernic acid and perlatolic acid. Fumarprotocetraric acid, 4-*O*-methylphysodic acid, 2′-*O*-methylphysodic acid and isophysodic acid could not be detected even as trace (Fig. 4C).

### Outcome of the technical validation

The implementation of the LDB into the feature-based molecular networking workflow allowed the identification of several metabolites expected in these lichens. Some additional molecules were also reported for the first time from these deeply-dug models. These seemingly surprising results may be related to formerly unreported chemosyndromic variations^7^ and/or to the higher sensitivity of this analytical strategy compared to formerly used techniques. Most hits were unique to the LDB, emphasizing the fact that most of these molecules were not reported before in MS/MS libraries of the GNPS. Metabolites absent from the LDB but detected in the studied lichens could be putatively identified, while completely unknown metabolites could be detected and linked to known scaffolds in lichens. These outcomes validate the adequacy of the LDB to dereplicate lichen metabolites. This network can be consulted on the GNPS platform at https://gnps.ucsd.edu/ProteoSAFe/status.jsp?task=ee1285c8de3a45cda13d719271570dc7.

## Supplementary information

Supporting Information.

## Data Availability

Data in .mzXML format was filtered and merged with an R script available at https://github.com/dolivierj/MSDB_Maker, using the R 3.6.0 language^63^ with the MSnbase^64^ package.

## References

[1] Honegger R (2000). Simon Schwendener (1829–1919) and the dual hypothesis of lichens. Bryologist.

[2] Perru O, Colin A, Perru O (2006). Aux origines des recherches sur la symbiose vers 1868–1883. Rev. Hist. Sci. Paris.

[3] Shibata S (2000). Yasuhiko Asahina (1880–1975) and his studies on lichenology and chemistry of lichen metabolites. Bryologist.

[4] Asahina, Y. In *Fortschritte Der Chemie Organischer Naturstoffe* 208–239 (Springer-Verlag, 1951).

[5] Asahina, Y. & Shibata, S. *Chemistry Of Lichen Substances*. (University of North Carolina Press, 1954).

[6] Culberson CF, Kristinsson H (1970). A standardized method for the identification of lichen products. J. Chromatogr..

[7] Culberson CF, Culberson WL (1976). Chemosyndromic variation in lichens. Syst. Bot..

[8] Culberson CF, Culberson WL (1978). *Cetrelia cetrarioides* and *C. monachorum* (Parmeliaceae) in the New World. Bryologist.

[9] Culberson CF, Culberson WL, Esslinger TL (1977). Chemosyndromic variation in the *Parmelia pulla* group. Bryologist.

[10] Stocker-Wörgötter E (2004). Secondary chemistry of lichen-forming fungi: chemosyndromic variation and DNA- analyses of cultures and chemotypes in the *Ramalina farinacea* complex. Bryologist.

[11] LaGreca S (1999). A phylogenetic evaluation of the *Ramalina americana* chemotype complex (Lichenized Ascomycota, Ramalinaceae) based on rDNA ITS sequence data. Bryologist.

[12] Elix JA, Crook CE (1992). The joint occurrence of chloroxanthones in lichens, and a further thirteen new lichen xanthones. Bryologist.

[13] Elix JA, Gaul KL (1986). The interconversion of the lichen depsides para- and meta-scrobiculin, and the biosynthetic implications. Aust. J. Chem..

[14] Elix JA, Jenie UA, Parker JL (1987). A novel synthesis of the lichen depsidones divaronic acid and stenosporonic acid, and the biosynthetic implications. Aust. J. Chem..

[15] Hale ME (1956). Chemical strains of the lichen *Parmelia furfuracea*. Am. J. Bot..

[16] Hale ME (1956). Fluorescence of lichen depsides and depsidones as a taxonomic criterion. Castanea.

[17] Culberson WL (1969). The use of chemistry in the systematics of the lichens. Taxon..

[18] Brodo IM (1986). Interpreting chemical variation in lichens for systematic purposes. Bryologist.

[19] Lumbsch, H. T. In *Chemical Fungal Taxonomy* (ed. Dekker, M.) 345–387 (CRC Press, 1998).

[20] Lumbsch, H. T. In *Protocols In Lichenology: Culturing, Biochemistry, Ecophysiology And Use In Biomonitoring* (eds. Kranner, I. C., Beckett, R. P. & Varma, A. K.) 281–295 (Springer Nature, 2002).

[21] Schmitt I, Lumbsch HT (2004). Molecular phylogeny of the Pertusariaceae supports secondary chemistry as an important systematic character set in lichen-forming ascomycetes. Mol. Phylogenet. Evol..

[22] Crespo A (2010). Phylogenetic generic classification of parmelioid lichens (Parmeliaceae, Ascomycota) based on molecular, morphological and chemical evidence. Taxon..

[23] Lumbsch HT (2014). High frequency of character transformations is phylogenetically structured within the lichenized fungal family Graphidaceae (Ascomycota: Ostropales). Syst. Biodivers..

[24] Huneck Siegfried, Yoshimura Isao (1996). Identification of Lichen Substances. Identification of Lichen Substances.

[25] Le Pogam P (2015). Matrix-free UV-laser desorption ionization mass spectrometry as a versatile approach for accelerating dereplication studies on lichens. Anal. Chem..

[26] Huovinen K, Hiltunen R, Von Schantz M (1982). A standardized HPLC method for analyses of lichen compounds from the genus. Cladonia. Planta Med.

[27] Yoshimura, I., Kinoshita, Y., Yamamoto, Y., Huneck, S. & Yamada, Y. Analysis of secondary metabolites from lichen by high performance liquid chromatography with a photodiode array detector. *Phytochem. Anal* **5**, 197–205 (1994).

[28] Le Pogam P, Le Lamer A, Legouin B, Boustie J, Rondeau D (2016). *In situ* DART-MS as a versatile and rapid dereplication tool in lichenology: chemical fingerprinting of *Ophioparma ventosa*. Phytochem. Anal.

[29] Le Corvec M (2016). Chemotaxonomic discrimination of lichen species using an infrared chalcogenide fibre optic sensor: a useful tool for on-field biosourcing. RSC Adv.

[30] Varol, M. In *Studies In Natural Products Chemistry*, Vol. 60 (ed. Atta-ur-Rahman) Ch. 12 (Elsevier B.V., 2018).

[31] Le Pogam, P., Herbette, G. & Boustie, J. In *Recent Advances In Lichenology*, Vol. 1 (eds. Upreti, D., Divakar, P., Shukla, V. & Bajpai, R) Ch. 11 (2015).

[32] Myllys L, Lindgren H, Aikio S, Häkkinen L, Högnabba F (2016). Chemical diversity and ecology of the genus *Bryoria* section *Implexae* (Parmeliaceae) in Finland. Bryologist.

[33] Gerlach A, da CL, Clerc P, Borges da Silveira RM (2017). Taxonomy of the corticolous, shrubby, esorediate, neotropical species of *Usnea* Adans. (Parmeliaceae) with an emphasis on southern Brazil. Lichenol..

[34] Jha BN (2017). Investigation of antioxidant, antimicrobial and toxicity activities of lichens from high altitude regions of Nepal. BMC Complement. Altern. Med..

[35] Ekman S, Tønsberg T (2019). *Biatora alnetorum* (Ramalinaceae, Lecanorales), a new lichen species from western North America. MycoKeys.

[36] Kalb K, Aptroot A (2018). New lichen species from Brazil and Venezuela. Bryologist.

[37] Kalb J, Lücking R, Kalb K (2018). The lichen genera *Allographa* and *Graphis* (Ascomycota: Ostropales, Graphidaceae) in Thailand—eleven new species, forty-seven new records and a key to all one hundred and fifteen species so far recorded for the country. Phytotaxa.

[38] Aptroot A (2018). & da Silva Caceres, M. E. New lichen species from Chapada Diamantina, Bahia, Brazil. Bryologist.

[39] Huneck S. (2001). New Results on the Chemistry of Lichen Substances. Fortschritte der Chemie organischer Naturstoffe / Progress in the Chemistry of Organic Natural Products.

[40] Elix, J. *A Catalogue Of Standardized Chromatographic Data And Biosynthetic Relationships For Lichen Substances*. (Self-Published, 2014).

[41] Le Pogam P, Boustie J (2016). Xanthones of lichen source: a 2016 update. Molecules.

[42] Schumm, F. & Elix, J. A. *Atlas Of Images Of Thin Layer Chromatograms Of Lichen Substances*. (Herstellung und Verlag: Books on Demand GmbH, 2015).

[43] Rambold G (2016). Geographic heat maps of lichen traits derived by combining LIAS light description and GBIF occurrence data, provided on a new platform. Biodivers. Conserv..

[44] Rambold G (2014). LIAS light – towards the ten thousand species milestone. MycoKeys.

[45] Mietzsch E, Lumbsch HT, Elix JA (1993). A new computer program for the identification on lichen substances. Mycotaxon.

[46] Crittenden PD, Porter N (1991). Lichen-forming fungi: potential sources of novel metabolites. Trends Biotechnol..

[47] Grube M (2019). Lichens — a promising source of bioactive secondary metabolites. Plant Genet. Resour.

[48] Molnár K, Farkas E (2010). Current results on biological activities of lichen secondary metabolites: a review. Zeitschrift für Naturforsch. C.

[49] Goga, M. *et al*. In *Co-Evolution Of Secondary Metabolites* (eds. Merillon, J.-M. & Ramawat, K. G.) 1–36 (Springer International Publishing, 2018).

[50] Schinkovitz A (2018). Secondary metabolites from lichen as potent inhibitors of advanced glycation end products and vasodilative agents. Fitoterapia.

[51] Boustie J, Tomasi S, Grube M (2011). Bioactive lichen metabolites: alpine habitats as an untapped source. Phytochem. Rev.

[52] Huneck S (1999). The significance of lichens and their metabolites. Naturwissenschaften.

[53] Stocker-Wörgötter E (2008). Metabolic diversity of lichen-forming ascomycetous fungi: culturing, polyketide and shikimate metabolite production, and PKS genes. Nat. Prod. Rep..

[54] Duong T (2018). Sanctis A – C: three racemic procyanidin analogues from the lichen *Parmotrema sancti-angelii*. European J. Org. Chem..

[55] Duong T (2018). Tsavoenones A-C: unprecedented polyketides with a 1,7-dioxadispiro[4.0.4.4]tetradecane core from the lichen *Parmotrema tsavoense*. Org. Biomol. Chem..

[56] Fox Ramos AE (2019). Collected mass spectrometry data on monoterpene indole alkaloids from natural product chemistry research. Sci. Data.

[57] Wang M (2016). Perspective sharing and community curation of mass spectrometry data with global natural products social molecular networking. Nat. Biotechnol..

[58] Haug K (2013). MetaboLights - an open-access general-purpose repository for metabolomics studies and associated meta-data. Nucleic Acids Res.

[59] Olivier-Jimenez D (2019). MetaboLights.

[60] Adusumilli, R. & Mallick, P. In *Proteomics: Methods and Protocols* (eds. Comai, L., Katz, J. E. & Mallick, P.) Ch. 23 (Springer Nature, 2017).

[61] Chambers MC (2012). A cross-platform toolkit for mass spectrometry and proteomics. Nat. Biotechnol..

[62] Kessner D, Chambers M, Burke R, Agus D, Mallick P (2008). ProteoWizard: open source software for rapid proteomics tools development. Bioinformatics.

[63] R Development Core Team. R: a language and environment for statistical computing. (2008).

[64] Gatto L, Lilley KS (2012). MSnbase-an R/bioconductor package for isobaric tagged mass spectrometry data visualization, processing and quantitation. Bioinformatics.

[65] Mann M (1990). Electrospray: its potential and limitations as an ionization method for biomolecules. Org. Mass Spectrom.

[66] Rafaëlly L, Héron S, Nowik W, Tchapla A (2008). Optimisation of ESI-MS detection for the HPLC of anthraquinone dyes. Dye. Pigment..

[67] Holcapek M, Volna K, Vanerkova D (2007). Effects of functional groups on the fragmentation of dyes in electrospray and atmospheric pressure chemical ionization mass spectra. Dye. Pigment..

[68] Pluskal T, Castillo S, Villar-Briones A, Orešič M (2010). MZmine 2: modular framework for processing, visualizing, and analyzing mass spectrometry-based molecular profile data. BMC Bioinformatics.

[69] Schymanski EL (2014). Identifying small molecules via high resolution mass spectrometry: communicating confidence. Environ. Sci. Technol..

[70] Böcker S, Letzel MC, Lipták Z, Pervukhin A (2009). SIRIUS: decomposing isotope patterns for metabolite identification. Bioinformatics.

[71] Holzmann G, Leuckert C (1990). Applications of negative fast atom bombardment and MS/MS to screening of lichen compounds. Phytochemistry.

[72] Le Pogam P (2016). Minor pyranonaphthoquinones from the apothecia of the lichen *Ophioparma ventosa*. J. Nat. Prod..

[73] Skult H (1997). Notes on the chemical and morphological variation of the lichen *Ophioparma ventosa* in East Fennoscandia. Ann. Bot. Fenn..

[74] May PF (1997). *Ophioparma lapponica*—a misunderstood species. Harvard Pap. Bot.

[75] Joulain D, Tabacchi R (2009). Lichen extracts as raw materials in perfumery. Part 1: oakmoss. Flavour Fragr. J..

[76] Culberson CF (1963). The lichen substances of the genus *Evernia*. Phytochemistry.

[77] Kosanić M, Manojlović N, Janković S, Stanojković T, Ranković B (2013). *Evernia prunastri* and *Pseudoevernia furfuraceae* lichens and their major metabolites as antioxidant, antimicrobial and anticancer agents. Food Chem. Toxicol..

[78] Molnár K, Farkas E (2011). Depsides and depsidones in populations of the lichen *Hypogymnia physodes* and its genetic diversity. Ann. Bot. Fenn..

[79] Latkowska E (2015). Phytochemistry secondary metabolites of the lichen *Hypogymnia physodes* (L.) Nyl. and their presence in spruce (*Picea abies* (L.) H. Karst.) bark. Phytochemistry.

[80] Białońska D, Dayan FE (2005). Chemistry of the lichen *Hypogymnia physodes* transplanted to an industrial region. J. Chem. Ecol..

[81] Solhaug KA, Lind M, Nybakken L, Gauslaa Y (2009). Possible functional roles of cortical depsides and medullary depsidones in the foliose lichen *Hypogymnia physodes*. Flora.

[82] Ranković B, Kosanić M, Manojlović N, Rančić A, Stanojković T (2014). Chemical composition of *Hypogymnia physodes* lichen and biological activities of some its major metabolites. Med. Chem. Res..

[83] Cansaran-Duman D, Cetin D, Sismek H, Coplu N (2010). Antimicrobial activities of the lichens *Hypogymnia vittata*, *Hypogymnia physodes* and *Hypogymnia tubulosa* and HPLC analysis of their usnic acid content. Asian J. Chem..

[84] Avalos A, Vicente C (1987). The occurrence of lichen phenolics in the photobiont cells of *Evernia prunastri*. Plant Cell Rep..

[85] Vicente C, Pérez-Urria E (1988). Tolbutamide, a urea derivative, impedes phenolic accumulation in the lichen *Evernia prunastri*. J. Plant Physiol..

[86] Herrero-Yudego P, Martin-Pedrosa M, Norato J, Vicente C (1989). Some features about usnic acid accumulation and its movement between the symbionts of the lichen, *Evernia prunastri*. J. Plant Physiol..

[87] Díaz-Guerra D, Manrique E (1984). Sustancias liquénicas en taxones de la provincia de Madrid I. *Evernia prunastri* (L.) Acb. y *Parmelina tiliacea* (Hoffm.) Hale. Lazaroa.

[88] Legaz ME (1986). Annual variations in arginine metabolism and phenolic content of *Evernia prunastri*. Environ. Exp. Bot..

[89] Bjerke JW, Lerfall K, Elvebakk A (2002). Effects of ultraviolet radiation and PAR on the content of usnic and divaricatic acids in two arctic-alpine lichens. Photochem. Photobiol. Sci..

