# Supplementary material for: A database of high-resolution MS/MS spectra for lichen metabolites
Source: Sci Data. 2019 Nov 28;6:294. doi: 10.1038/s41597-019-0305-1 (PMC6882832; doi:10.1038/s41597-019-0305-1)
Supplement: Supplementary file 1 — Supporting Information. [file 41597_2019_305_MOESM1_ESM.docx]

**Supporting Information**

**A database of high-resolution MS/MS spectra for lichen metabolites**

### Authors

Damien Olivier-Jimenez^1,2^, Marylène Chollet-Krugler^1^, David Rondeau^2,3^, Mehdi A. Beniddir^4^, Solenn Ferron^1^, Thomas Delhaye^2^, Pierre-Marie Allard^5^, Jean-Luc Wolfender^5^, Harrie J. M. Sipman^6^, Robert Lücking^6^, Joël Boustie^1,*^ and Pierre Le Pogam^4,*^.

**Affiliations**

1. CNRS, ISCR (Institut des Sciences Chimiques de Rennes)-UMR 6226, Univ Rennes, F-35000 Rennes, France

2. CNRS, IETR (Institut d'Électronique et Télécommunications de Rennes)-UMR 6164, Univ Rennes, F-35000 Rennes, France

3. Département de Chimie, Université de Bretagne Occidentale, F-29238 Brest, France

4. CNRS, BioCIS (Biomolécules : Conception Isolement et Synthèse)-UMR 8076, Univ Paris-Sud, Université Paris-Saclay, 5, rue J.-B. Clément F-92290 Châtenay-Malabry, France

5. School of Pharmaceutical Sciences, EPGL, University of Geneva, University of Lausanne, CMU, 1 Rue Michel Servet, 1211 Geneva 4, Switzerland

6. Botanischer Garten und Botanisches Museum, Freie Universität Berlin, Königin-Luise-Strasse 6–8, D-14195 Berlin, Germany

Corresponding author(s): [joel.boustie@univ-rennes1.fr](mailto:joel.boustie@univ-rennes1.fr); [pierre.le-pogam-alluard@u-psud.fr](mailto:pierre.le-pogam-alluard@u-psud.fr).

**Table of Contents**

**Supplementary Table S1**. Parameters used to produce the molecular networks using the spectra of the LDB seen in Fig. 2 **2**

**Supplementary Table S2**. Lichens used for technical validation **2**

**Supplementary Table S3**. Workflow used in MZmine and parameters used in order to process the raw LC-MS/MS data from the three lichens to produce a molecular network **3**

**Supplementary Table S4**. Parameters used to produce the molecular network for the three studied lichens, shown in Fig. 3 **4**

**Supplementary Figure S1**. Mirror view of a spectrum from *O. ventosa* putatively identified as nordivaricatic acid against divaricatic acid **4**

**Supplementary Figure S2**. Mirror view of a spectrum from *O. ventosa* at *m/z* 479.0596 against usnic acid **5**

**Supplementary Figure S3**. Mirror view of a spectrum from *E. prunastri* at *m/z* 347.0764 against evernic acid **6**

**Supplementary Figure S4**. Mirror view of a spectrum from *E. prunastri* at *m/z* 365.0442 against evernic acid **7**

**Supplementary Figure S5**. Mirror view of a spectrum from *H. physodes* putatively identified as conphysodalic against physodalic acid **8**

**Supplementary Table S5**. List of the lichen metabolites forming the LDB **9**

**References** **33**

**Molecular networks of the LDB spectra.** Each spectrum of the LDB was submitted to the GNPS to produce two molecular networks (negative and positive modes) represented in Fig. **2**. Parameters used are presented in Table **S1**.

**Table S1**. Parameters used to produce the molecular networks using the spectra of the LDB presented in Fig. **2**.

| Parameter | Value |
| --- | --- |
| Minimum pairs cosine | 0.6 |
| Parent mass ion tolerance | 0.02 |
| Fragment ion mass tolerance | 0.02 |
| Minimum matched fragment ions | 6 |
| Top K | 10 |
| Minimum cluster size | 1 |
| Maximum connected component size | 0 |
| Run MSCluster | No |
| Library search score threshold | 0.6 |
| Library search minimum matched peaks | 6 |

**Molecular network of *Ophioparma ventosa*, *Evernia prunastri,* and *Hypogymnia physodes* (technical validation).** Files resulting from the analysis of the three lichens (Table **S2**) in the .d format were converted using MSConvert in the .mzXML format and were imported in MZmine 2^1^. The pre-processing parameters used in MZmine 2 are described in Table S2. The resulting peak list was then converted to an .mgf format to be submitted to the GNPS server and produce a molecular network. Parameters for this network are exposed in Table S3.

**Table S2**. Lichens used for technical validation.

| Lichen | Rennes herbarium Ref. | Collection details | Collector, identification |
| --- | --- | --- | --- |
| *Evernia prunastri* (L.) Ach. | JB/13/156 | On oak tree Jayac, Périgord (France), 170 m (01/2013)  N 45.031'486’’  E 1.36’156 | Joël Boustie, Rennes |
| *Ophioparma ventosa* (L.) Norman | JB/14/211 | On siliceous rock, lake Großer  Winterleitensee, Styria (Austria), 2000 m  N 47.005’15’’  E 14.33’45’’ | Walter Obermayer, Graz |
| *Hypogymnia physodes* (L.) Nyl. | JB/18/234 | On the bark of resinous trees, Liffré forest, near Rennes (France), 110 m  N 48.12’32.5’’  W1.33’24.3’’ | Damien Olivier, Rennes |

Table **S3**. MZmine 2 pre-processing parameters used in order to process the raw LC-MS/MS data from the three lichens

| Algorithm | Parameters |
| --- | --- |
| Mass detection | MS1: 1.0E3 MS2: 5.0E1 |
| ADAP Chromatogram builder^2^ | Min. group size: 1 Group intensity threshold: 1.0E3 Min highest intensity: 1.0E3 *m/z* tolerance: 20 ppm |
| Chromatogram deconvolution (ADAP Wavelets^2^) | *m/z* range for MS2 scan pairing (DA): 0.3 RT range for MS2 scan pairing (min): 1 S/N threshold: 2 S/N estimator: Intensity window SN min feature height: 1000 coefficient/area threshold: 5 Peak duration range: 0.01-7.00 RT wavelet range: 0.01-0.50 |
| Isotopic peaks grouper | *m/z* tolerance: 20 ppm Retention time tolerance: 0.2 min Monotonic shape: unchecked Maximum charge: 2 Representative isotope: Most intense |
| Join aligner | *m/z* tolerance: 20 ppm Weight for *m/z*: 1 Retention time tolerance: 0.7 Weight for RT: 1 Require same charge state: unchecked Require same ID: unchecked |
| Same RT and *m/z* range gap-filler | *m/z* tolerance: 20 ppm |
| Peak list rows filter | Only checked values: Keep only peaks with MS2 scan (GNPS) Reset the peak number ID |

**Table S4**. Parameters used to produce the molecular network for the three lichens, shown in Fig. **3**.

| Parameter | Value |
| --- | --- |
| Minimum pairs cosine | 0.6 |
| Parent mass ion tolerance | 0.02 |
| Fragment ion mass tolerance | 0.02 |
| Minimum matched fragment ions | 6 |
| Top K | 10 |
| Minimum cluster size | 1 |
| Maximum connected component size | 0 |
| Run MSCluster | No |
| Library search score threshold | 0.5 |
| Library search minimum matched peaks | 4 |

**Data on unannotated nodes from the network in Figure 3.** The spectra of metabolites absent from the LDB are compared with closely related metabolites to assess their hypothetical identity with MZmine’s mirror view of MS/MS data. Molecular formulas were calculated using the SIRIUS software on MS^1^ spectra of each compound. Spectra were imported to SIRIUS in an MGF spectra with a [M+?]^-^ ion. Computation was carried out with a unlimited number of atoms from C, H, N, O. Instrument selected was a Q-TOF, mass error of 10 ppm, top 10 candidates were calculated).


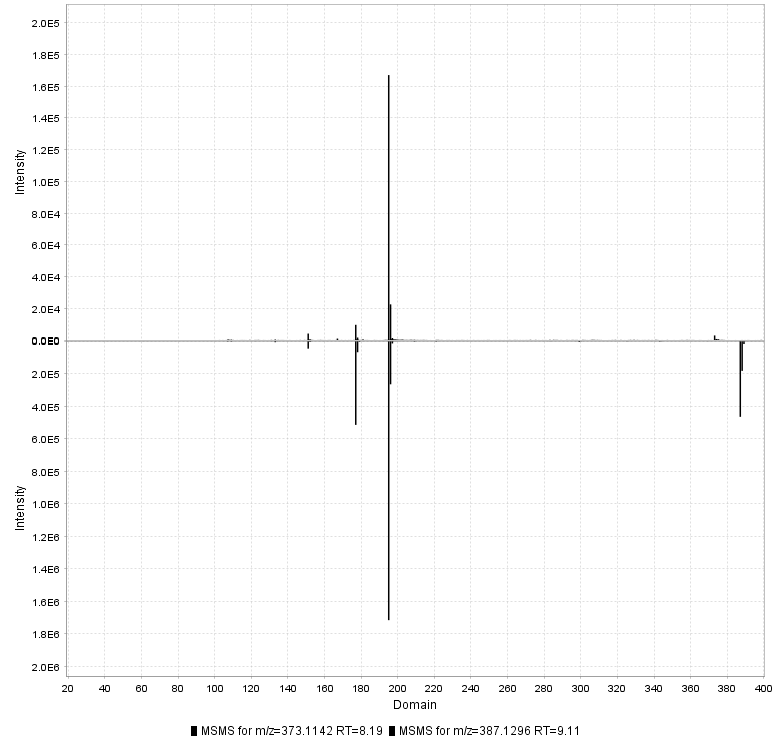


373.1330

195.0668

177.0552

151.0760

387.1478

195.0675

177.0566

151.0770


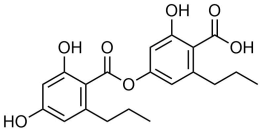

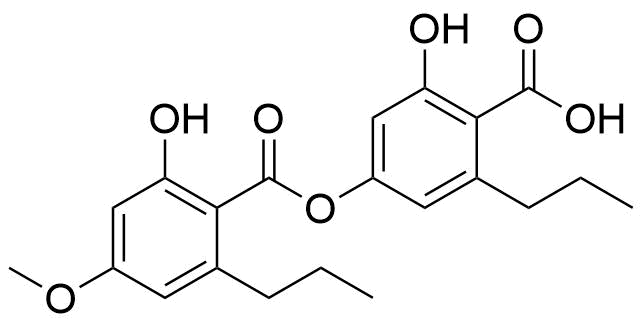


UNK 373
Nordivaricatic acid?
C_20_H_22_O_7_
Th. *m/z* [M-H]^-^ 373.1292
Exp. *m/z* 373.1315 (-6.0 ppm)

Divaricatic acid
C_21_H_24_O_7_
Th*. m/z* [M-H]^-^ 387.1449

?

**Fig. S1**. Mirror view of a spectrum from *O. ventosa* putatively identified as nordivaricatic acid against divaricatic acid. Calculated molecular formula is consistent with that of nordivaricatic acid.


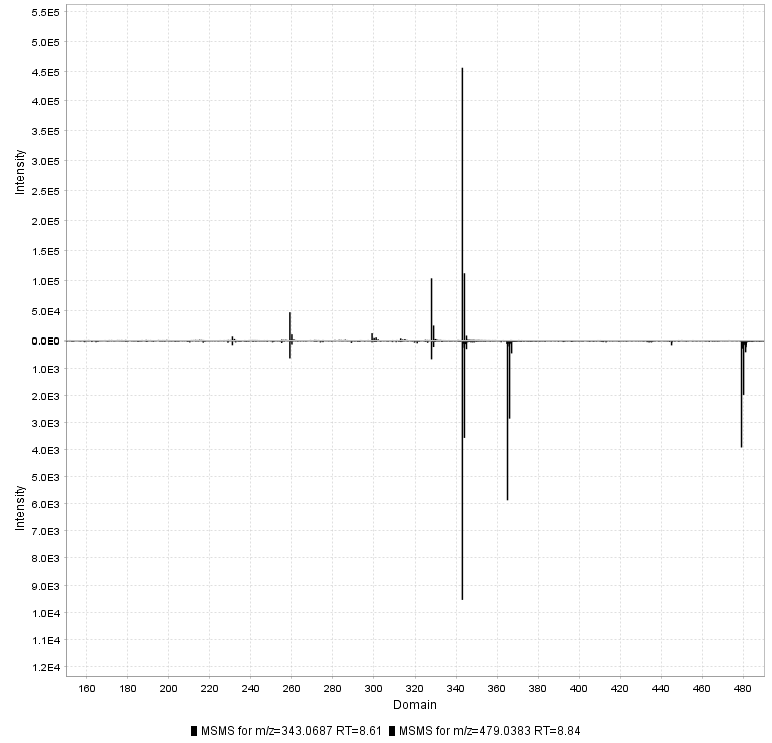


479.0571

365.0653

343.0836

328.0615

259.0605

231.0630

231.0664

259.0615

299.0930

328.0596

343.0840


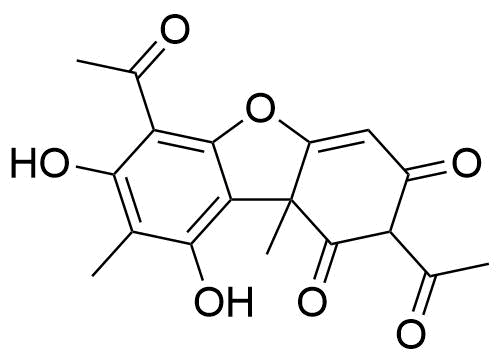


Usnic acid
C_18_H_16_O_7_
Th. *m/z* [M-H]^-^ 343.0822

UNK 479
C_24_H_16_O_11_
Th. *m/z* [M-H] 479.0619
Exp *m/z* [M-H]^-^ 479.0596 (5.0 ppm)

**Fig. S2**. Mirror view of a spectrum from *O. ventosa* at *m/z* 479.0596 against usnic acid.


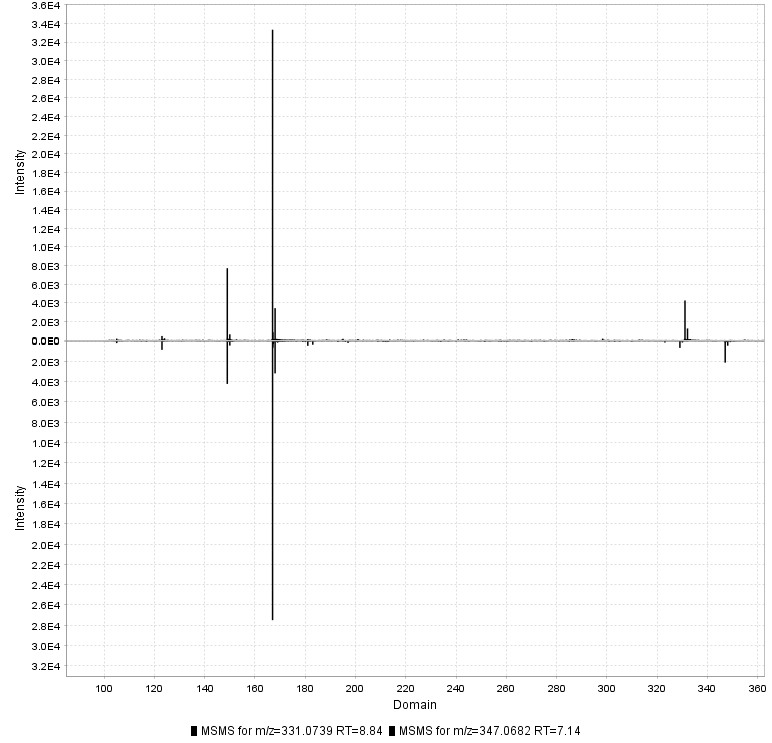


UNK 347
C_17_H_16_O_8_
Th. *m/z* [M-H] 347.0771
Exp. *m/z* 347.0764 (2.5 ppm)

347.0750

329.0629

167.0343

149.0229

123.0446

105.0342

331.0825

167.0346

149.0242

123.0440

105.0312


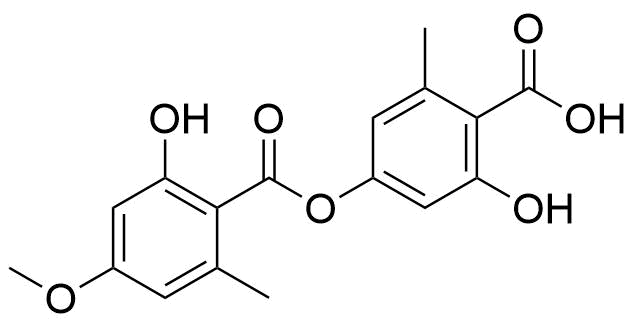


Evernic acid
C_17_H_16_O_7_
Th. *m/z* [M-H]^-^ 331.0823

**Fig. S3**. Mirror view of a spectrum from *E. prunastri* at *m/z* 347.0764 against evernic acid. Calculated molecular formula suggests an additional hydroxyl function on the molecule compared to evernic acid.


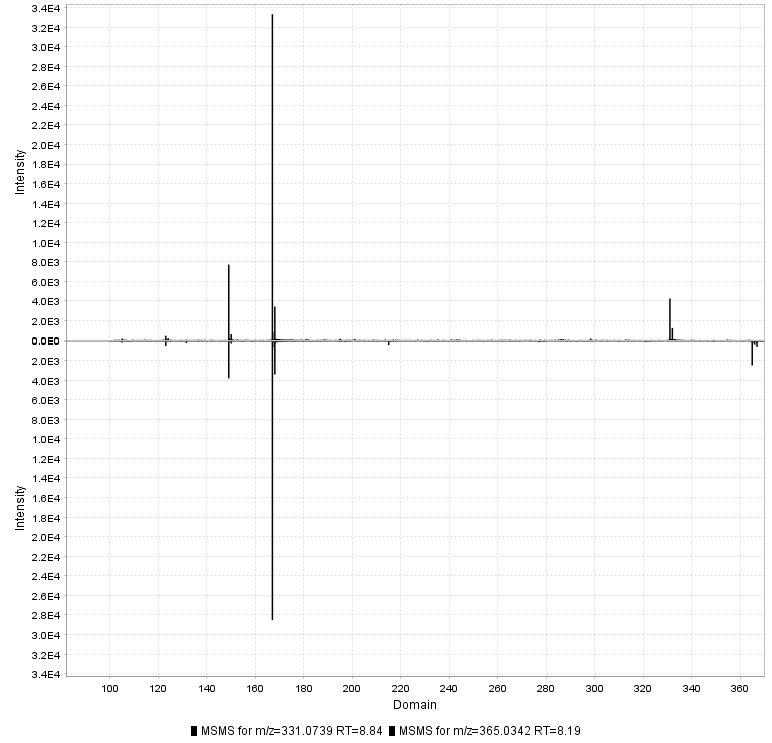

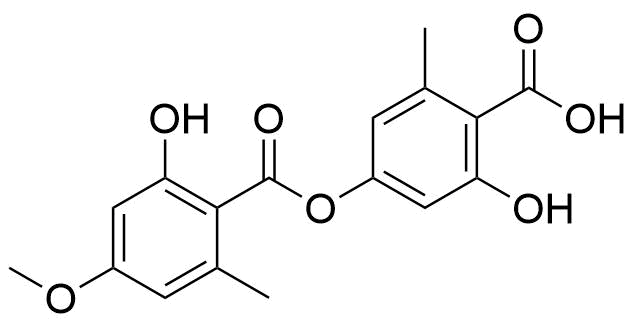


Evernic acid
C_17_H_16_O_7_
*m/z* [M-H]^-^ 331.0818

331.0825

167.0346

149.0242

123.0440

105.0312

365.0431

167.0353

149.0243

123.0444

105.0380

UNK 365
C_17_H_14_O_7_
Th. *m/z* [M+Cl]^-^ 365.0443
Exp *m/z* 365.0442 (-0.2 ppm)

**Fig. S4**. Mirror view of a spectrum from *E. prunastri* at *m/z* 365.0442 against evernic acid. Calculated molecular formula suggests a molecule similar to evernic acid with an addition double bond equivalent.


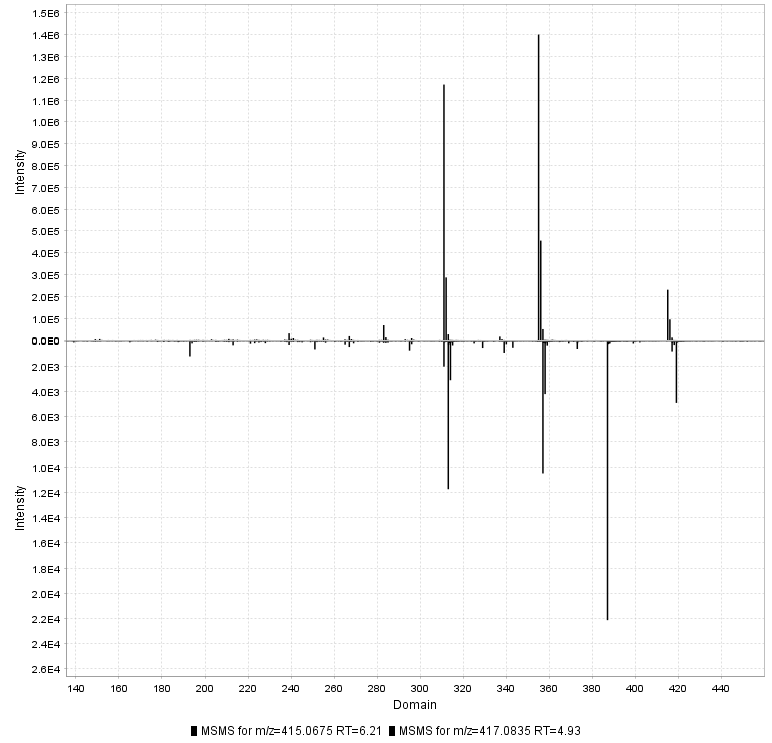

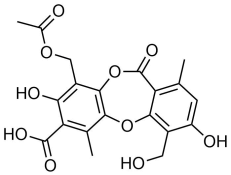

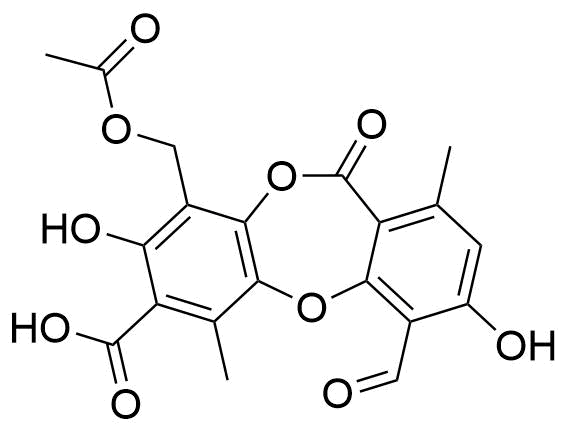


Physodalic acid
C_20_H_16_O_10_
Th. *m/z* [M-H]^-^ 415.0670

UNK 417
Conphysodalic acid?
C_20_H_18_O_10_
Th.*m/z* [M-H] 417.0827
Exp. *m/z* 417.0833 (-1.5 ppm)

387.0699

357.0611

313.0708

417.0861

415.0691

355.0484

311.0583

?

**Fig. S5**. Mirror view of a spectrum from *H. physodes* putatively identified as conphysodalic against physodalic acid. Chemical formula was calculated with the *m/z* value as an [M-H]^-^ adduct. Calculated chemical formula is consistent with that of conphysodalic acid.

**Metabolites represented in the LDB.** A list of the metabolites analyzed by LC-MS/MS and uploaded to form the LDB is presented hereafter in Table S4 with names, structural classes, exact masses, retention times in minutes and structures.

**Table S5**. List of the lichen metabolites forming the LDB

| Substance | Structural class | Exact mass | Retention time (min) | Structure |
| --- | --- | --- | --- | --- |
| Acaranoic acid | Acids | 298.2144 | 3.77 | 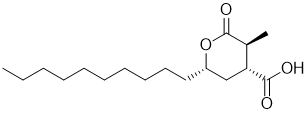 |
| Bourgeanic acid | Acids | 386.3032 | 0.46 | 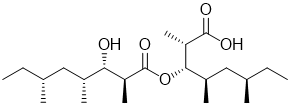 |
| Caperatic acid | Acids | 402.2618 | 5.84 | 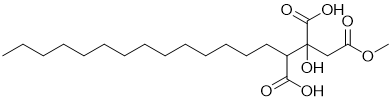 |
| Fumaric acid | Acids | 116.011 | 5.25 | 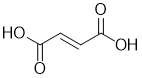 |
| Isorangiformic acid | Acids | 386.2668 | 6.9 | 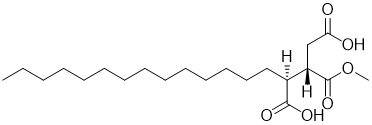 |
| Lichesterylic acid | Acids | 298.2508 | 9.58 | 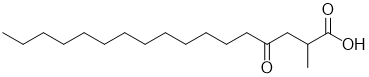 |
| Norrangiformic acid | Acids | 372.2512 | 8.03 | 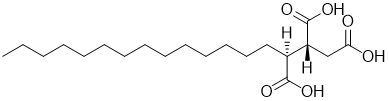 |
| Rangiformic acid | Acids | 386.2668 | 6.35 | 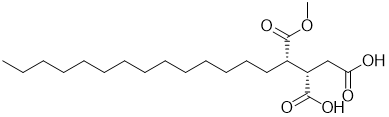 |
| Roccellic acid | Acids | 300.2301 | 7.83 | 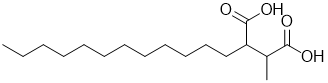 |
| Acetylportentol | Aliphatic and Cycloaliphatic Compounds | 352.1886 | 8.23 | 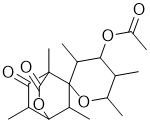 |
| Aspicilin | Aliphatic and Cycloaliphatic Compounds | 328.225 | 8.19 | 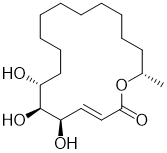 |
| Lepranthin | Aliphatic and Cycloaliphatic Compounds | 660.3357 | 0.28 | 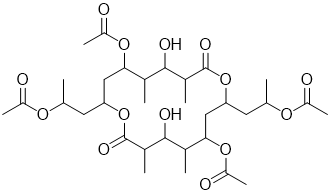 |
| Portentol | Aliphatic and Cycloaliphatic Compounds | 310.178 | 4.51 | 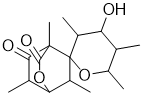 |
| Alectorialic acid | Benzyldepsides | 376.0794 | 5.12 | 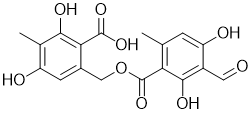 |
| Barbatolic acid | Benzyldepsides | 390.0587 | 5.61 | 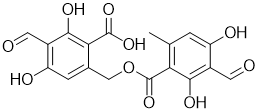 |
| 2,4-Dichlor-1,3,6-tri-O-methylnorlichexanthone | Chromanes and Chromones | 368.0218 | 4.52 | 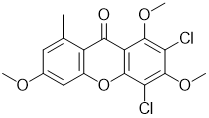 |
| 2,8-Dimethyl-5,7-dimethoxychromone | Chromanes and Chromones | 234.0892 | 4.15 | 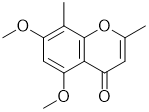 |
| 5,7-Dihydroxy-2,6,8-trimethylchromone | Chromanes and Chromones | 220.0736 | 4.7 | 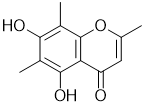 |
| Acetylloboridin | Chromanes and Chromones | 536.153 | 5.06 | 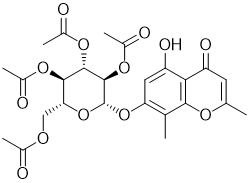 |
| Eugenitin | Chromanes and Chromones | 220.0736 | 5.27 | 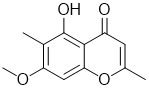 |
| Eugenitol | Chromanes and Chromones | 206.0579 | 6.89 | 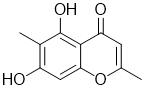 |
| Galapagin | Chromanes and Chromones | 424.1369 | 7.53 | 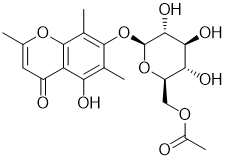 |
| Isoeugenitol | Chromanes and Chromones | 206.0579 | 3.83 | 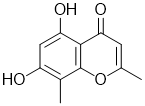 |
| Lepraric acid | Chromanes and Chromones | 362.1002 | 4.69 | 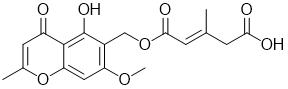 |
| Lobodirin | Chromanes and Chromones | 494.1424 | 6.72 | 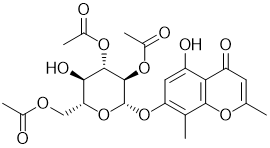 |
| Mollin | Chromanes and Chromones | 410.1213 | 5.25 | 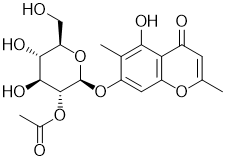 |
| Roccellin | Chromanes and Chromones | 452.1319 | 5.98 | 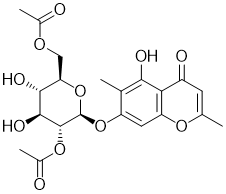 |
| Rupicolin (sordidone) | Chromanes and Chromones | 240.0189 | 2.35 | 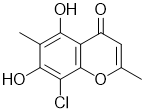 |
| 2,4,6-Trihydroxyacetophenone | Cleavage Products of Depsides and Depsidones | 168.0423 | 4.54 | 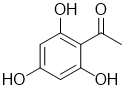 |
| 2,4-Di-O-methylolivetonide | Cleavage Products of Depsides and Depsidones | 276.1362 | 6.74 | 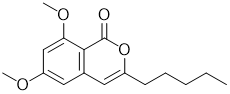 |
| 3,5-Dichlororsellinic acid | Cleavage Products of Depsides and Depsidones | 235.9643 | 7.26 | 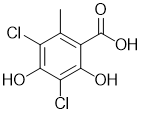 |
| 4-O-methylolivetolcarboxylic acid | Cleavage Products of Depsides and Depsidones | 238.1205 | 7.48 | 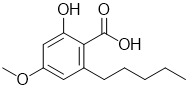 |
| 5-Chlorodivaricatinic acid | Cleavage Products of Depsides and Depsidones | 244.0502 | 7.28 | 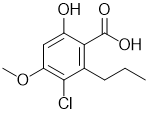 |
| 5-Chloroorsellinic acid | Cleavage Products of Depsides and Depsidones | 202.0033 | 6.03 | 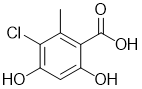 |
| Atranol | Cleavage Products of Depsides and Depsidones | 152.0473 | 7.14 | 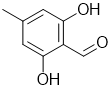 |
| Beta-orcinol | Cleavage Products of Depsides and Depsidones | 138.0681 | 6.35 | 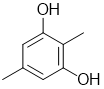 |
| b-Orcinolcarboxylic acid | Cleavage Products of Depsides and Depsidones | 182.0579 | 7.26 | 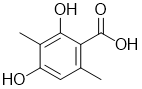 |
| Chloratranol | Cleavage Products of Depsides and Depsidones | 186.0084 | 7.27 | 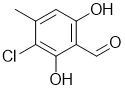 |
| Divaric acid | Cleavage Products of Depsides and Depsidones | 196.0736 | 7.09 | 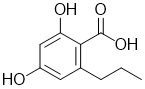 |
| Divaricatinic acid | Cleavage Products of Depsides and Depsidones | 210.0892 | 4.69 | 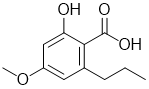 |
| Divarinol | Cleavage Products of Depsides and Depsidones | 152.0837 | 4.52 | 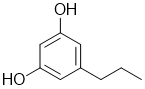 |
| Glomellin | Cleavage Products of Depsides and Depsidones | 234.0892 | 6 | 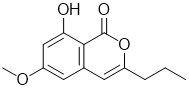 |
| Haematommic acid | Cleavage Products of Depsides and Depsidones | 196.0372 | 6.92 | 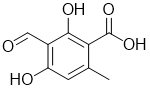 |
| Isoevernic acid | Cleavage Products of Depsides and Depsidones | 182.0579 | 7.28 | 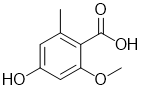 |
| Methyl beta-orcinolcarboxylate | Cleavage Products of Depsides and Depsidones | 196.0736 | 6.37 | 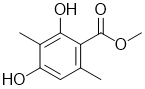 |
| Methyl Haematommate | Cleavage Products of Depsides and Depsidones | 210.0528 | 7.47 | 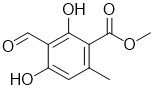 |
| Methyl orsellincarboxylate | Cleavage Products of Depsides and Depsidones | 182.0579 | 5.3 | 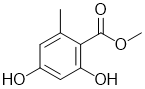 |
| Olivetolcarboxylic acid | Cleavage Products of Depsides and Depsidones | 224.1049 | 6.84 | 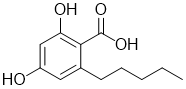 |
| Olivetonide | Cleavage Products of Depsides and Depsidones | 248.1049 | 6.73 | 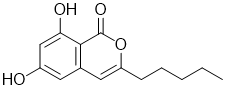 |
| Orsellinic acid | Cleavage Products of Depsides and Depsidones | 168.0423 | 6.74 | 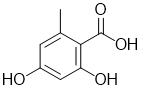 |
| Rhizonic acid | Cleavage Products of Depsides and Depsidones | 196.0736 | 10.43 | 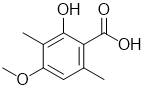 |
| 3,5-Dichloro-2-O-methylanziaic acid | Depsides (Didepsides) | 512.1369 | 7.52 | 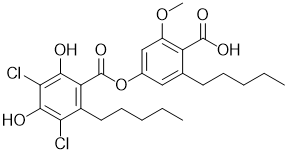 |
| 3-Chlorodivaricatic acid | Depsides (Didepsides) | 422.1132 | 7.84 | 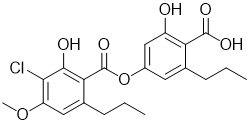 |
| 5-chlorodivaricatic acid | Depsides (Didepsides) | 422.1132 | 7.11 | 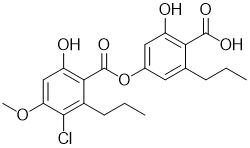 |
| Anziaic acid | Depsides (Didepsides) | 430.1992 | 7.83 | 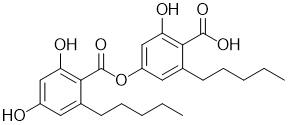 |
| Arthoniaic acid | Depsides (Didepsides) | 528.2359 | 8.76 | 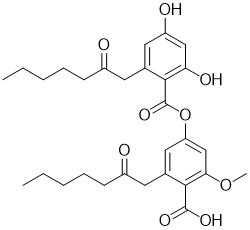 |
| Atranorin | Depsides (Didepsides) | 374.1002 | 5.79 | 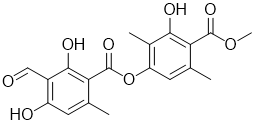 |
| Baeomycesic acid | Depsides (Didepsides) | 374.1002 | 8.19 | 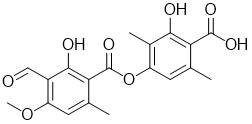 |
| Barbatic acid | Depsides (Didepsides) | 360.1209 | 6.07 | 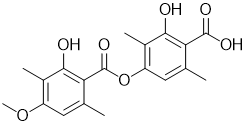 |
| Chloroatranorin | Depsides (Didepsides) | 408.0612 | 7.37 | 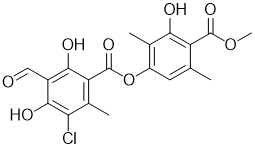 |
| Confluentic acid | Depsides (Didepsides) | 500.241 | 7.47 | 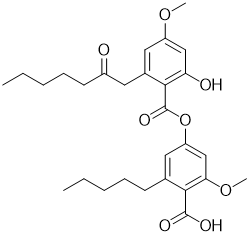 |
| Cryptochlorophaeic acid | Depsides (Didepsides) | 460.2097 | 6.54 | 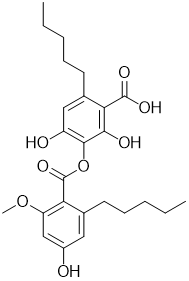 |
| Diffractaic acid | Depsides (Didepsides) | 374.1366 | 7.33 | 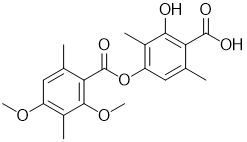 |
| Diploschistesic acid | Depsides (Didepsides) | 334.0689 | 7.31 | 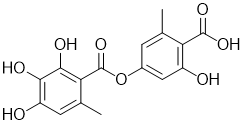 |
| Divaricatic acid | Depsides (Didepsides) | 388.1522 | 5.8 | 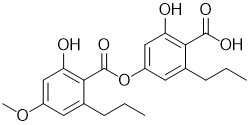 |
| Erythrin | Depsides (Didepsides) | 422.1213 | 5.63 | 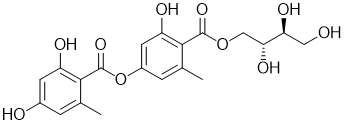 |
| Evernic acid | Depsides (Didepsides) | 332.0896 | 6.2 | 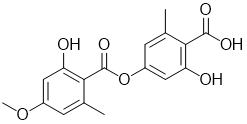 |
| Glomellic acid | Depsides (Didepsides) | 472.1733 | 6.17 | 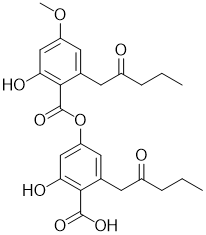 |
| Glomelliferic acid | Depsides (Didepsides) | 458.1941 | 6.53 | 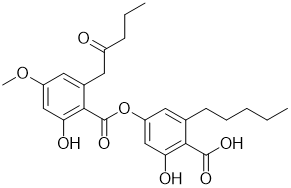 |
| Haemathamnolic acid | Depsides (Didepsides) | 404.0743 | 7.34 | 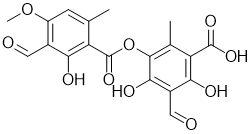 |
| Imbricaric acid | Depsides (Didepsides) | 416.1835 | 7.28 | 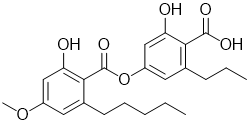 |
| Lecanoric acid | Depsides (Didepsides) | 318.074 | 7.27 | 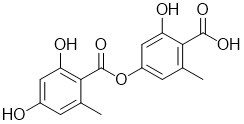 |
| Merochlorophaeic acid | Depsides (Didepsides) | 446.1941 | 7.13 | 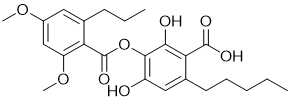 |
| Miriquidic acid | Depsides (Didepsides) | 458.1941 | 10.9 | 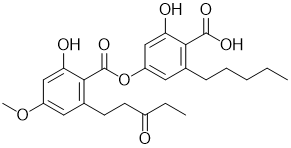 |
| Nephroarctin | Depsides (Didepsides) | 372.1209 | 4.06 | 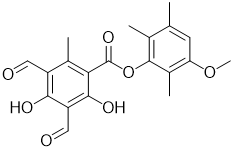 |
| Obtusatic acid | Depsides (Didepsides) | 346.1053 | 6.91 | 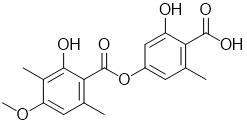 |
| Olivetoric acid | Depsides (Didepsides) | 472.2097 | 7.46 | 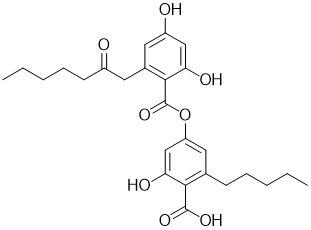 |
| Perlatolic acid | Depsides (Didepsides) | 444.2148 | 7.35 | 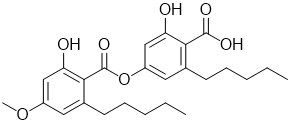 |
| Planaic acid | Depsides (Didepsides) | 472.2461 | 6.04 | 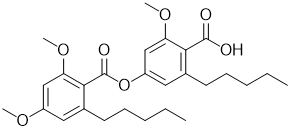 |
| Pseudocyphellarin A | Depsides (Didepsides) | 402.1315 | 6.99 | 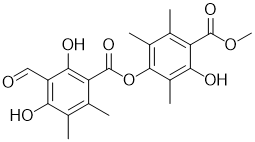 |
| Scrobiculin | Depsides (Didepsides) | 418.1628 | 6.95 | 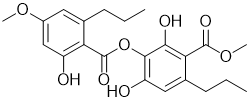 |
| Sekikaic acid | Depsides (Didepsides) | 418.1628 | 8.6 | 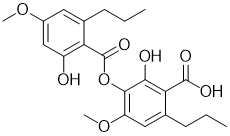 |
| Sphaerophorin | Depsides (Didepsides) | 416.1835 | 5.27 | 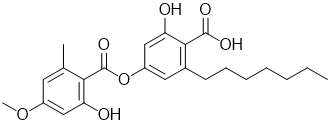 |
| Squamatic acid | Depsides (Didepsides) | 390.0951 | 6.23 | 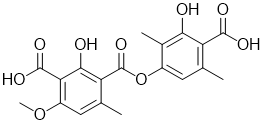 |
| Stenosporic acid | Depsides (Didepsides) | 416.1835 | 7.27 | 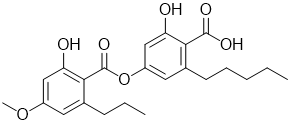 |
| Thamnolic acid | Depsides (Didepsides) | 420.0693 | 7.28 | 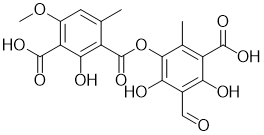 |
| Decarboxythamnolic acid | Depsides (Didepsides) | 376.0794 | 7.28 |  |
| Tumidulin | Depsides (Didepsides) | 400.0117 | 5.62 | 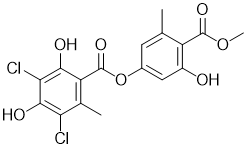 |
| Crustinic acid | Depsides (Tridepsides) | 484.1006 | 4.93 | 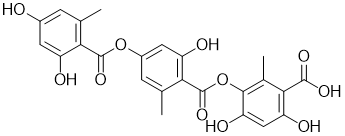 |
| Gyrophoric acid | Depsides (Tridepsides) | 468.1056 | 6.28 |  |
| Ovoic acid | Depsides (Tridepsides) | 482.1213 | 4.58 |  |
| Umbilicaric acid | Depsides (Tridepsides) | 482.1213 | 6.35 |  |
| 3-Dechloro-4-O-methyldiploicin | Depsidones | 401.9829 | 8.74 |  |
| 3-Hydroxycolensoic acid | Depsidones | 458.1941 | 10.45 |  |
| 3-Hydroxyphysodic acid | Depsidones | 486.189 | 7.64 |  |
| 3-Methoxycolensoic acid | Depsidones | 472.2097 | 4.49 |  |
| 9'-methylprotocetraric acid | Depsidones | 388.0794 | 6.36 |  |
| Alpha-Alectoronic acid | Depsidones | 512.2046 | 7.36 |  |
| Alpha-collatolic acid | Depsidones | 526.2203 | 6.62 |  |
| Argopsin | Depsidones | 396.0167 | 5.63 |  |
| Caloploicin | Depsidones | 401.9829 | 7.13 |  |
| Cetraric acid | Depsidones | 402.0951 | 5.08 |  |
| Chlorovirensic acid | Depsidones | 392.0299 | 4.51 |  |
| Colensoic acid | Depsidones | 442.1992 | 7.48 |  |
| Constictic acid | Depsidones | 402.0587 | 5.37 |  |
| Dechlorodiploicin | Depsidones | 387.9672 | 7.09 |  |
| Diploicin | Depsidones | 421.9282 | 7.45 |  |
| Eriodermin | Depsidones | 382.0011 | 7.4 |  |
| Fumarprotocetraric acid | Depsidones | 472.0642 | 3.95 |  |
| Gangaleoidin | Depsidones | 412.0117 | 5.63 |  |
| Hypoprotocetraric acid | Depsidones | 344.0896 | 4.72 |  |
| Leoidin | Depsidones | 412.0117 | 7.27 |  |
| Lividic acid | Depsidones | 500.2046 | 4.01 |  |
| Lobaric acid | Depsidones | 456.1784 | 6.88 |  |
| Nornotatic acid | Depsidones | 330.074 | 7.08 |  |
| Norstictic acid | Depsidones | 372.0481 | 5.81 |  |
| Notatic acid | Depsidones | 344.0896 | 7.1 |  |
| Pannarin | Depsidones | 362.0557 | 10.21 |  |
| Physodalic acid | Depsidones | 416.0743 | 10.02 |  |
| Physodic acid | Depsidones | 470.1941 | 5.82 |  |
| Protocetraric acid | Depsidones | 374.0638 | 4.01 |  |
| Psoromic acid | Depsidones | 358.0689 | 4.33 |  |
| Salazinic acid | Depsidones | 388.043 | 3.25 |  |
| Scensidin | Depsidones | 368.0218 | 8.46 |  |
| Stictic acid | Depsidones | 386.0638 | 3.99 |  |
| Succinprotocetraric acid | Depsidones | 474.0798 | 5.24 |  |
| Variolaric acid | Depsidones | 314.0427 | 5.97 |  |
| Virensic acid | Depsidones | 358.0689 | 3.41 |  |
| Picrolichenic acid | Depsones | 442.1992 | 5.09 |  |
| Didymic acid | Dibenzofuranes | 370.178 | 9.12 |  |
| Isousnic acid | Dibenzofuranes | 344.0896 | 0.28 |  |
| Pannaric acid | Dibenzofuranes | 316.0583 | 5.25 |  |
| Pannarol | Dibenzofuranes | 228.0786 | 4.15 |  |
| Placodiolic acid | Dibenzofuranes | 376.1158 | 5.98 |  |
| Porphyrilic acid | Dibenzofuranes | 314.0427 | 6.19 |  |
| Pseudoplacodiolic acid | Dibenzofuranes | 376.1158 | 7.07 |  |
| Schizopeltic acid | Dibenzofuranes | 358.1053 | 5.16 |  |
| Strepsilin | Dibenzofuranes | 270.0528 | 6.82 |  |
| Usnic acid | Dibenzofuranes | 344.0896 | 5.08 |  |
| Beta-Collatolic acid | Diphenylethers | 526.2203 | 6.6 |  |
| Buellin | Diphenylethers | 434.0091 | 6.17 |  |
| Lobariolcarboxylic acid (Lobarin) | Diphenylethers | 474.189 | 5.5 |  |
| Mycosporine glutamicol | Mycosporines | 303.1318 | 7.09 |  |
| Mycosporine glutaminol | Mycosporines | 302.1478 | 8.74 |  |
| Mycosporine serinol | Mycosporines | 261.1212 | 6.93 |  |
| Simonyellin | Naphthopyranes | 274.0477 | 6.9 |  |
| Arthonin | N-Containing Compounds | 424.2362 | 8.1 |  |
| Hypothallin | N-Containing Compounds | 472.2362 | 8.75 |  |
| Isoarthonin | N-Containing Compounds | 424.2362 | 6.53 |  |
| Roccanin | N-Containing Compounds | 488.2424 | 7.09 |  |
| Solorinin | N-Containing Compounds | 297.1576 | 8 |  |
| allo-Pertusaric acid | Paraconic acids | 366.2406 | 7.82 |  |
| allo-Protolichesterinic acid | Paraconic acids | 324.2301 | 7.46 |  |
| Dihidroprotolichesterinic acid | Paraconic acids | 326.2457 | 7.46 |  |
| Dihydromuronic acid | Paraconic acids | 368.2563 | 6.72 |  |
| Dihydropertusaric | Paraconic acids | 368.2563 | 7.1 |  |
| Isomurolic acid | Paraconic acids | 368.2563 | 8 |  |
| Isomuronic acid | Paraconic acids | 366.2406 | 7.45 |  |
| Lichesterinic acid | Paraconic acids | 324.2301 | 8 |  |
| Murolic acid | Paraconic acids | 368.2563 | 7.26 |  |
| Muronic acid | Paraconic acids | 366.2406 | 7.27 |  |
| Neodihydroprotolichesterinic acid | Paraconic acids | 326.2457 | 6.71 |  |
| Nephromopsic acid | Paraconic acids | 326.2457 | 9.71 |  |
| Nephrosteranic acid | Paraconic acids | 298.2144 | 7.49 |  |
| Pertusaric acid | Paraconic acids | 366.2406 | 7.68 |  |
| Protolichesterinic acid | Paraconic acids | 324.2301 | 7.82 |  |
| Roccellaric acid | Paraconic acids | 326.2457 | 6.75 |  |
| Arabitol | Polyols, Monosaccharides, Carbohydrates | 152.0685 | 7.64 |  |
| D-Mannitol | Polyols, Monosaccharides, Carbohydrates | 182.079 | 7.65 |  |
| Mesoeryhthritol | Polyols, Monosaccharides, Carbohydrates | 122.0579 | 6.95 |  |
| Volemitol | Polyols, Monosaccharides, Carbohydrates | 212.0896 | 6.08 |  |
| Calycin | Pulvinic Acid Derivatives | 306.0528 | 6.4 |  |
| Epanorin | Pulvinic Acid Derivatives | 435.1682 | 5.07 |  |
| Leprapinic acid | Pulvinic Acid Derivatives | 352.0947 | 5.07 |  |
| Norepanorin | Pulvinic Acid Derivatives | 421.1525 | 6.16 |  |
| Norrhizocarpic acid | Pulvinic Acid Derivatives | 455.1369 | 5.29 |  |
| Pinastric acid | Pulvinic Acid Derivatives | 352.0947 | 4.75 |  |
| Pulvinic acid | Pulvinic Acid Derivatives | 308.0685 | 5.98 |  |
| Pulvinic acid dilactone | Pulvinic Acid Derivatives | 290.0579 | 5.48 |  |
| Rhizocarpic acid | Pulvinic Acid Derivatives | 469.1525 | 6.9 |  |
| Vulpinic acid | Pulvinic Acid Derivatives | 322.0841 | 6.71 |  |
| Acetylhaemoventosine | Quinones | 346.0689 | 5.62 |  |
| Canarione | Quinones | 272.0321 | 5.68 |  |
| Canarione dimethylether | Quinones | 300.0634 | 6.58 |  |
| Chiodectonic acid | Quinones | 334.0325 | 6.81 |  |
| Chrysophanol | Quinones | 254.0579 | 8.37 |  |
| Citreorosein | Quinones | 286.0477 | 3.96 |  |
| Emodic acid | Quinones | 300.027 | 4.14 |  |
| Emodin | Quinones | 270.0528 | 4.5 |  |
| Endocrocin | Quinones | 314.0427 | 4.34 |  |
| Erythroglaucin | Quinones | 300.0634 | 4.52 |  |
| Fallacinal | Quinones | 298.0477 | 5.62 |  |
| Fallacinol (Teloschistin) | Quinones | 300.0634 | 4.63 |  |
| Fragilin | Quinones | 318.0295 | 8.39 |  |
| Haematommone | Quinones | 314.0427 | 9.11 |  |
| Haemoventosine | Quinones | 304.0583 | 1.62 |  |
| Hydroxyhaemoventosine | Quinones | 320.0532 | 9.4 |  |
| Methoxyhaemoventosine | Quinones | 334.0689 | 8.79 |  |
| Parietin | Quinones | 284.0685 | 8.95 |  |
| Parietinic acid | Quinones | 314.0427 | 9.16 |  |
| Polyporic acid | Quinones | 292.0736 | 9.25 |  |
| Rhodocladonic acid | Quinones | 318.0376 | 8.93 |  |
| Rugulosin | Quinones | 542.1213 | 8.47 |  |
| Skyrin | Quinones | 538.09 | 8.62 |  |
| Solorinic acid | Quinones | 384.1209 | 7.89 |  |
| Thelephoric acid | Quinones | 352.0219 | 6.35 |  |
| Xanthorin | Quinones | 300.0634 | 7.3 |  |
| Isokaurane | Terpenoids : Diterpenes | 272.2504 | 5.43 |  |
| Kaurane | Terpenoids : Diterpenes | 272.2504 | 5.81 |  |
| 12-Desoxydiacetylpyxinol | Terpenoids : Triterpenes | 544.4128 | 6 |  |
| 17(21)-Hopen-3-one | Terpenoids : Triterpenes | 424.3705 | 5.09 |  |
| 17(21)-Hopen-6-one | Terpenoids : Triterpenes | 424.3705 | 6.35 |  |
| 21-Hopen-3-one | Terpenoids : Triterpenes | 424.3705 | 6.73 |  |
| 22a-Acetoxy-3-stictanone | Terpenoids : Triterpenes | 484.3916 | 6.72 |  |
| 22-Hydoxy-2-hopen-1-one | Terpenoids : Triterpenes | 440.3654 | 6.21 |  |
| 22-Hydroxy-1-hopanone | Terpenoids : Triterpenes | 442.3811 | 6.91 |  |
| 22-hydroxy-6-hopanone | Terpenoids : Triterpenes | 442.3811 | 6.73 |  |
| 22-hydroxy-7-hopanone | Terpenoids : Triterpenes | 442.3811 | 6.36 |  |
| 25-Acetoxy-3b-hydroxy-20(S),24(R)-epoxydammarane | Terpenoids : Triterpenes | 502.4022 | 5.47 |  |
| 2a-Acetoxy-3,22-stictandione | Terpenoids : Triterpenes | 498.3709 | 7.67 |  |
| 6a-Acetoxy-17(21)-hopene | Terpenoids : Triterpenes | 468.3967 | 5.8 |  |
| Diacetylpyxinol | Terpenoids : Triterpenes | 560.4077 | 6.35 |  |
| Ursolic acid | Terpenoids : Triterpenes | 456.3603 | 6.2 |  |
| 1,3,6-tri-O-methylarthothelin | Xanthones and bis-Xanthones | 401.9829 | 7.09 |  |
| 1,6-Dihydroxy-3,8-dimethylxanthone | Xanthones and bis-Xanthones | 256.0736 | 5.61 |  |
| 2,4,5-Trichloronorlichexanthone | Xanthones and bis-Xanthones | 354.0062 | 6.94 |  |
| 2,4-Dichlor-3,6-di-O-methylnorlichexanthone | Xanthones and bis-Xanthones | 354.0062 | 6.25 |  |
| 2,4-Dichlorolichexanthone | Xanthones and bis-Xanthones | 325.9749 | 4.05 |  |
| 2,4-Dichloronorlichexanthone | Xanthones and bis-Xanthones | 387.9672 | 7.27 |  |
| 2,5,7-trichlorolichexanthone | Xanthones and bis-Xanthones | 368.0218 | 7.82 |  |
| 2,5-Dichlor-1,3,6-tri-O-methylnorlichexanthone | Xanthones and bis-Xanthones | 354.0062 | 5.99 |  |
| 2,5-Dichlorolichexanthone | Xanthones and bis-Xanthones | 325.9749 | 0.3 |  |
| 2,5-Dichloronorlichexanthone | Xanthones and bis-Xanthones | 306.0295 | 5.06 |  |
| 2-Chloro-6-O-methylnorlichexanthone | Xanthones and bis-Xanthones | 292.0139 | 3.97 |  |
| 2-Chloronorlichexanthone | Xanthones and bis-Xanthones | 306.0295 | 4.51 |  |
| 4-Chloro-6-O-methylnorlichexanthone | Xanthones and bis-Xanthones | 320.0452 | 6.72 |  |
| 4-Chlorolichexanthone | Xanthones and bis-Xanthones | 292.0139 | 5.48 |  |
| 4-Chloronorlichexanthone | Xanthones and bis-Xanthones | 373.9516 | 5.3 |  |
| 6-O-Methylarthothelin | Xanthones and bis-Xanthones | 292.0139 | 6.54 |  |
| 7-Chloronorlichexanthone | Xanthones and bis-Xanthones | 359.9359 | 7.88 |  |
| Arthothelin | Xanthones and bis-Xanthones | 286.0841 | 6.72 |  |
| Lichexanthone | Xanthones and bis-Xanthones | 258.0528 | 7.09 |  |
| Norlichexanthone | Xanthones and bis-Xanthones | 638.1636 | 0.4 |  |
| Secalonic acid A | Xanthones and bis-Xanthones | 638.1636 | 0.46 |  |
| Secalonic acid B | Xanthones and bis-Xanthones | 638.1636 | 0.67 |  |
| Secalonic acid D | Xanthones and bis-Xanthones | 638.1636 | 6.94 |  |
| Secalonic acid F | Xanthones and bis-Xanthones | 393.8969 | 6 |  |
| Thiophanic acid | Xanthones and bis-Xanthones | 339.9905 | 5.81 |  |
| Thiophaninic acid | Xanthones and bis-Xanthones | 373.9516 | 6.01 |  |
| Thuringione | Xanthones and bis-Xanthones | 306.0295 | 4.23 |  |
| Vinetorin | Xanthones and bis-Xanthones | 298.2144 | 4.32 |  |

**References**

1. Pluskal, T., Castillo, S., Villar-Briones, A. & Orešič, M. MZmine 2: modular framework for processing, visualizing, and analyzing mass spectrometry-based molecular profile data. *BMC Bioinformatics* **11**, 11 (2010).

2. Myers, O. D., Sumner, S. J., Li, S., Barnes, S. & Du, X. One step forward for reducing false positive and false negative compound identifications from mass spectrometry metabolomics data: new algorithms for constructing extracted ion chromatograms and detecting chromatographic peaks. *Anal. Chem.* **89**, 8696–8703 (2017).
